# Loss of m^6^A Methyltransferase METTL5 Promotes Cardiac Hypertrophy Through Epitranscriptomic Control of SUZ12 Expression

**DOI:** 10.3389/fcvm.2022.852775

**Published:** 2022-02-28

**Authors:** Yanchuang Han, Tailai Du, Siyao Guo, Lu Wang, Gang Dai, Tianxin Long, Ting Xu, Xiaodong Zhuang, Chen Liu, Shujuan Li, Dihua Zhang, Xinxue Liao, Yugang Dong, Kathy O. Lui, Xu Tan, Shuibin Lin, Yili Chen, Zhan-Peng Huang

**Affiliations:** ^1^Department of Cardiology, Center for Translational Medicine, Institute of Precision Medicine, The First Affiliated Hospital, Sun Yat-sen University, Guangzhou, China; ^2^NHC Key Laboratory of Assisted Circulation, Sun Yat-sen University, Guangzhou, China; ^3^Department of Nephrology, The First Affiliated Hospital, Sun Yat-sen University, Guangzhou, China; ^4^Department of Chemical Pathology, Li Ka Shing Institute of Health Sciences, The Chinese University of Hong Kong, Prince of Wales Hospital, Shatin, Hong Kong SAR, China; ^5^School of Pharmaceutical Sciences, Center for Infectious Disease Research, School of Medicine, Tsinghua University, Tsinghua-Peking Center for Life Sciences, Beijing, China

**Keywords:** cardiac hypertrophy, METTL5, RNA modification, translational regulation, SUZ12

## Abstract

Enhancement of protein synthesis from mRNA translation is one of the key steps supporting cardiomyocyte hypertrophy during cardiac remodeling. The methyltransferase-like5 (METTL5), which catalyzes m^6^A modification of 18S rRNA at position A_1832_, has been shown to regulate the efficiency of mRNA translation during the differentiation of ES cells and the growth of cancer cells. It remains unknown whether and how METTL5 regulates cardiac hypertrophy. In this study, we have generated a mouse model, METTL5-cKO, with cardiac-specific depletion of METTL5 *in vivo*. Loss function of METTL5 promotes pressure overload-induced cardiomyocyte hypertrophy and adverse remodeling. The regulatory function of METTL5 in hypertrophic growth of cardiomyocytes was further confirmed with both gain- and loss-of-function approaches in primary cardiomyocytes. Mechanically, METTL5 can modulate the mRNA translation of SUZ12, a core component of PRC2 complex, and further regulate the transcriptomic shift during cardiac hypertrophy. Altogether, our study may uncover an important translational regulator of cardiac hypertrophy through m6A modification.

## Introduction

The cardiac plasticity refers to the capability of the heart to remodel in response to environmental demands. One of the cardiac responses to mechanical or pathological stress is cardiac hypertrophy (1). Hypertrophic growth of cardiomyocytes, in which the size and contractile force of cardiomyocytes increase significantly, is one of the major cellular changes in response to stress. It has been well-studied that hypertrophic growth involves control of cardiomyocyte gene expression at multiple molecular levels (2, 3). Multiple signal transduction pathways, such as PI3K/AKT (4) and MAPK (5), and transcriptional factors, such as MEF2 (6), NFAT (7), and GATA4 (8), have been demonstrated to control the shift of transcriptome toward hypertrophy. Enhanced protein synthesis and sarcomere assembly are main features of hypertrophic growth of cardiomyocytes. The observation that transcriptome data deviate from mass spectrum data (9) indicates that translational control is a key step of regulating this cellular process. Recently, our study suggests that the increased quantity of ribosomes is one of the main contributors of enhanced protein synthesis in cardiomyocyte hypertrophy (10). However, how cardiac hypertrophy is controlled at the step of translation remains largely unknown.

Methylation is one of the well-known modifications in ribonucleic acid, including m^6^A, m^5^C, m^1^A, m^7^G, and m^6^Am (11). These modified nucleic acids widely exist in ribosomal RNAs, transfer RNAs, messenger RNAs and even some non-coding RNAs (12). Enzymes responsible for the dynamics of these RNA modifications have also been explored. For example, the METTL3/METTL14/WTAP protein complex is responsible for adding a methyl group to the targeted adenosine in RNAs (13, 14), whereas demethylases, such as FTO (15) and ALKBH5 (16), erase the existing m^6^A modification from RNA molecules. In addition, multiple proteins, including YTHDC1, YTHDC2, YTHDF1, YTHDF2, and YTHDF3, have been identified as “readers,” which recognize and bind to m6A-modifed RNAs, and promote different processes, such as mRNA export from nucleus, mRNA degradation and mRNA translation (17). Two m^6^A RNA modification readers, YTHDF1 and YTHDF3, have been shown to promote the translational efficiency of their target mRNAs by interacting with ribosome 40S and 60S subunits and translation initiation factor complex 3 (18, 19). These studies indicate m^6^A methylation could play an important role in regulating gene expression at the step of translation. Indeed, alteration of m^6^A methylation leads to the change of protein abundance in an mRNA-level-independent manner during heart failure (20). The importance of translational control in cardiac remodeling and cardiomyocyte hypertrophy indicates m^6^A RNA methylation dynamics is critical to the pathogenesis of cardiac disease. Supportively, recent work has demonstrated that manipulation of cardiac m^6^A modification level by either depleting m^6^A “writer” METTL3 (21) or overexpressing “eraser” FTO (22) regulates cardiac disease progression in different pathological conditions.

METTL5 is a methyltransferase containing a typical SAM-binding motif and a conserved m^6^A-catalyzing NPPF motif (23). Recent studies have demonstrated that METTL5 forms a complex with TRMT112 and catalyzes methylation of 18S rRNA m^6^A_1832_ (23– 25). METTL5 has been shown to regulate the differentiation of embryonic stem (ES) cells (24, 26) and the growth of cancer cells (27). Furthermore, mechanistic studies link the function of METTL5 to the regulation of mRNA translation by possibly fine-tuning the interaction between ribosome decoding center and translating mRNA through methylation of 18S rRNA (27). However, the functional role of METTL5 in cardiac physiology and pathology has not yet been studied. In this study, we take advantage of a genetic model, in which the function of METTL5 is abolished in a cardiomyocyte-specific manner, to study its function in the heart. We show that METTL5 promotes stress-induced cardiac hypertrophy mainly via regulating the translation of SUZ12 mRNA. Altogether, our study may uncover an important translational mechanism for maintaining the homeostats of heart cells via epitranscriptomic regulation.

## Results

### The Expression Profile of METTL5 and Its Physiological Function in the Heart

**Figure 1:**
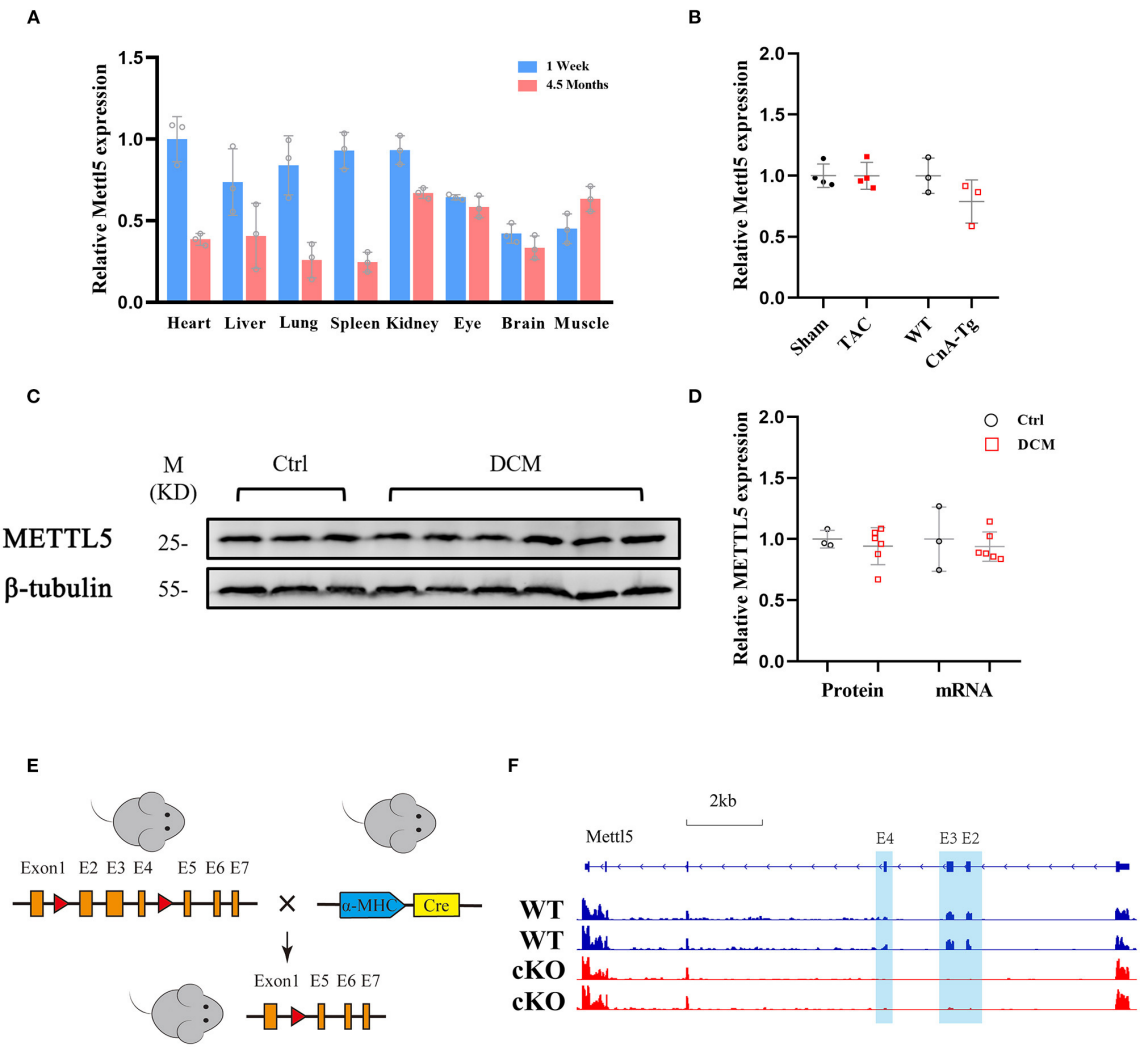
METTL5 expression profile and the generation of METTL5-cKO mouse. **(A)** Relative gene expression of METTL5 in different organs from 1 week and 4.5 months old mice by RT-PCR. *n* = 3 mice in each group. **(B)** Relative gene expression of METTL5 in TAC vs. Sham (*n* = 3 mice in each group) and CnA-Tg vs. WT (*n* = 4 mice in each group) mouse hearts by RT-PCR. **(C)** The protein level of METTL5 in control patient hearts (*n* = 3) and dilated cardiomyopathy (DCM) patient hearts (*n* = 6) by western blotting. β-tubulin srerves as a control. **(D)** Quantification of western blotting in panel C and the relative expression of METTL5 in control patient hearts (*n* = 3) and DCM patient hearts (*n* = 6) by RT-PCR. **(E)** Schematic of generation of METTL5 cardiomyocyte-specific knockout (METTL5-cKO) mouse. **(F)** The genomic view of reads in RNA-seq for METTL5 in the IGV browser.

Thus far, little is known about how N^6^-methyladenosine (m^6^A) modification of 18S rRNA at position A_1832_, which has been widely reported to be mediated by methyltransferase METTL5 (23–27), regulates cardiac physiology and/or pathology. To understand its function in the heart, we first examined its expression levels in different organs. Multiple mouse organs, including the heart, liver, lung, spleen, kidney, eye, brain and skeletal (Sk.) muscle, were collected from the neonatal (1 week) and adult (4.5 months) stages. METTL5 was detected as ubiquitously expressed in all tissues examined (Figure 1A). We then asked whether the expression level of METTL5 alters under pathological conditions. As a result, the expression level of METTL5 was reduced in Calcineurin transgenic (CnA-Tg) hearts but remained unaltered in transverse aortic binding (TAC)-induced cardiac hypertrophy (Figure 1B). In addition, the expression level of METTL5 in human samples collected from patients with heart failure was also tested (Supplementary Table 1). The mRNA and protein levels of METTL5 tended to decrease in the human failing hearts, respectively (Figures 1C,D).

METTL5 knockout (KO) mice were reported viable recently by our group and others (24, 26). Cardiac histological and transcriptomic analyses of 1-month-old METTL5-KO mice and their control littermates showed no obvious cardiac abnormality (Supplementary Figures 1, 2 and Supplementary Table 2), indicating that METTL5 is dispensable for heart development. To specifically study the function of METTL5 in cardiomyocytes *in vivo*, Myh6-Cre was crossed with METTL5^fl/fl^, in which exons 2, 3 and 4 are floxed, to generate the cardiac-specific METTL5 depleted (METTL5-cKO) mice (Figure 1E). The 3 months old METTL5-cKO mouse hearts showed no obvious abnormality by echocardiography and histological examination (Supplementary Figure 3 and Supplementary Table 3). Similarly, transcriptomic analyses only detected 28 genes with significant difference in METTL5-cKO hearts (Supplementary Table 4). Of note, the floxed exons 2–4 of METTL5 were almost undetectable in METTL5-cKO hearts, indicating that METTL5 was predominately expressed in cardiomyocytes (Figure 1F).

### Cardiac-Specific Ablation of METTL5 Promotes Stress-Induced Cardiac Remodeling

**Figure 2:**
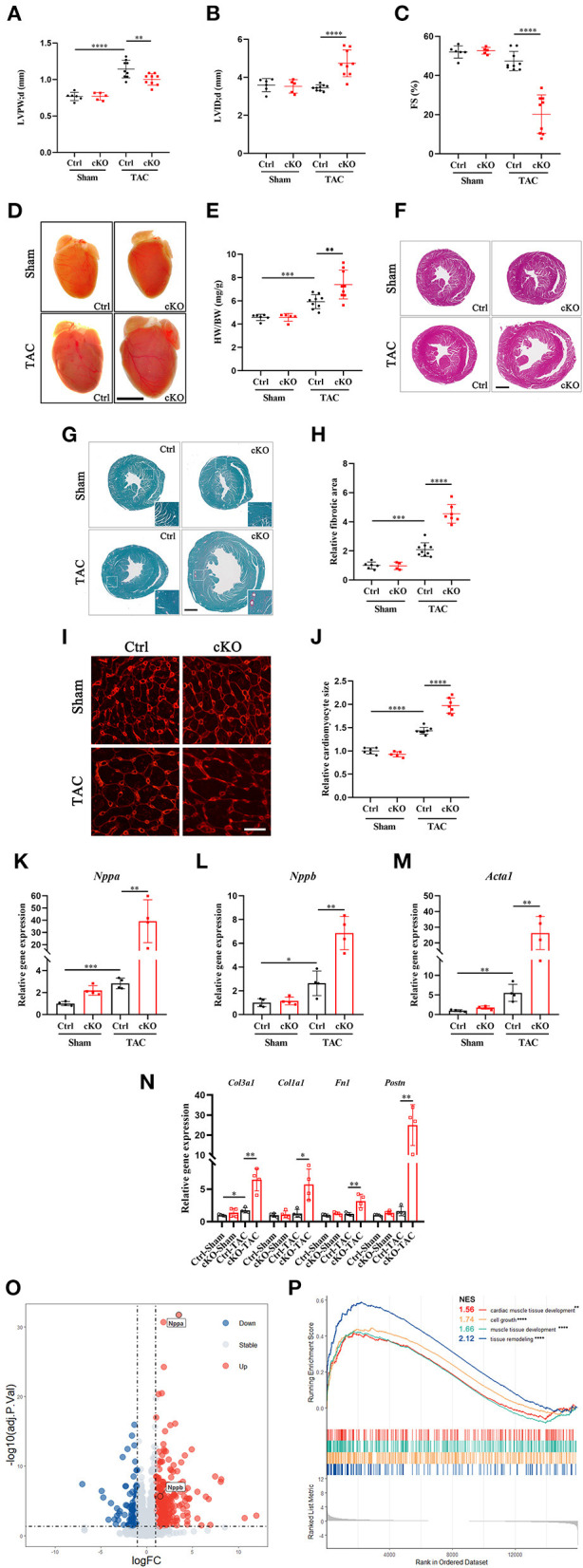
Cardiac-specific knockout of METTL5 *in vivo* promotes TAC-induced cardiac remodeling. **(A–C)** Left ventricular posterior wall thickness at end-diastole (LVPW;d), Left ventricular internal dimension at end-diastole (LVID;d), and Fractional shortening (FS) of TAC or sham operated METTL5-cKO and control mice at 4 weeks after surgery. *N* number (*n* > 4) for each group is indicated by the number of dots. **(D)** Representative images of gross morphology of METTL5-cKO and control hearts 4 weeks after TAC or sham operation. Scale bar = 5 mm. **(E)**. Quantification of heart weight to body weight ratio (HW/BW) of METTL5-cKO and control mice 4 weeks after TAC or sham operation. *N* number (*n* > 4) for each group is indicated by the number of dots. **(F)** Representative images of H&E staining of METTL5-cKO and control hearts 4 weeks after TAC or sham operation. Scale bar = 1 mm. **(G)** Representative images of Fast green and Sirius red staining of METTL5-cKO and control hearts 4 weeks after TAC or sham operation. Scale bar = 1 mm. **(H)** The fibrotic area of images from Fast green and Sirius red staining is quantified. *N* number (*n* > 3) for each group is indicated by the number of dots. **(I)** Heart cross sections were stained with wheat germ agglutinin (WGA). Scale bar = 25 μm. **(J)** Cardiomyocyte cross-sectional area was quantified. More than 500 cardiomyocytes from five hearts are quantified for each group. **(K–M)** Relative gene expression of hypertrophy marker genes by RT-PCR. *n* = 4 mice in each group. N. Relative gene expression of fibrosis related genes by RT-PCR. *n* = 4 mice in each group. **(O)** Volcano plot of differentially expressed genes in METTL5-cKO and control hearts 4 weeks after TAC operation. **(P)** GSEA analysis of differentially expressed genes in METTL5-cKO and control hearts 4 weeks after TAC operation. (The unpaired *T*-test was used for 2-group comparisons. *P < 0.05, **P < 0.01, ***P < 0.001, ****P < 0.0001).

In order to understand the function of METTL5 in cardiac remodeling, METTL5-cKO mice and control littermates were subjected to TAC surgery, which induced pressure overload in the left ventricle. As expected, echocardiographic examination showed significant increase in left ventricular posterior wall thickness (LVPW) without obvious chamber dilation and decrease in cardiac function in control mice at 4 weeks post-TAC (Figures 2A,B and Table 1). In contrast, loss of METTL5 promoted TAC-induced cardiac remodeling with progression to heart failure in the METTL5-cKO group, which was associated with significantly increased left ventricular internal dimension (LVID) and dramatic reduced fraction shortening (FS) (Figures 2B,C and Table 1). Hearts were collected at 4 weeks post-TAC after echocardiographic examination. The ratio of ventricular weight vs. body weight (Vw vs. Bw) and gross morphology consistently demonstrated the enlargement of METTL5-cKO hearts in the TAC group compared to that of the control hearts (Figures 2D,E). In addition, histological examination showed the obvious chamber dilation of METTL5-cKO TAC hearts (Figure 2F). Adverse cardiac remodeling and heart failure are often associated with increased cardiac fibrosis. Indeed, Sirius red/Fast green staining showed significantly increased fibrosis in TAC control hearts, which was further increased in METTL5-cKO TAC hearts (Figures 2G,H). In line with the further progression of cardiac remodeling in METTL5-cKO TAC group, Wheat Germ Agglutinin (WGA) staining indicated further hypertrophic growth of cardiomyocytes, which was evident by the cross area of cardiomyocytes, in METTL5-cKO TAC hearts compared to that of the control TAC hearts (Figures 2I,J).

**Table 1:** Echocardiography examination of METTl5-cKO mice and their control littermates with transverse aortic constriction (TAC) or sham operation at 4 weeks after surgery.

	**Ctrl; Sham (*N* = 6)**	**METTL5-cKO; Sham (*N* = 5)**	**Ctrl; TAC (*N* = 9)**	**METTL5-cKO; TAC (*N* = 9)**
IVS;d (mm)	0.732 ± 0.053	0.752 ± 0.022	1.145 ± 0.085^**^	1.004 ± 0.079^##^
IVS;s (mm)	1.362 ± 0.033	1.415 ± 0.148	1.789 ± 0.145^**^	1.345 ± 0.191^##^
LVID;d (mm)	3.590 ± 0.345	3.526 ± 0.353	3.452 ± 0.152	4.741 ± 0.706^##^
LVID;s (mm)	1.728 ± 0.233	1.672 ± 0.203	1.816 ± 0.199	3.834 ± 1.009^##^
LVPW;d (mm)	0.768 ± 0.056	0.769 ± 0.051	1.143 ± 0.120^**^	1.002 ± 0.078^##^
LVPW;s (mm)	1.413 ± 0.059	1.416 ± 0.117	1.733 ± 0.124^**^	1.346 ± 0.218^##^
EF (%)	83.83 ± 2.96	84.51 ± 1.61	79.53 ± 4.60	40.40 ± 17.75^##^
FS (%)	52.01 ± 3.16	52.64 ± 1.71	47.43 ± 4.90	20.30 ± 9.83^##^
LV Mass (mg)	91.01 ± 17.39	89.93 ± 16.93	156.84 ± 24.75^**^	212.53 ± 46.00^##^
LV Mass (Corrected, mg)	72.81 ± 13.91	71.94 ± 13.55	125.47 ± 19.80^**^	170.02 ± 36.80^##^
LV Vol;d (uL)	54.73 ± 11.90	52.42 ± 12.24	49.35 ± 5.25	107.42 ± 36.59^##^
LV Vol;s (uL)	8.99 ± 2.78	8.22 ± 2.45	10.14 ± 2.81	69.25 ± 41.25^##^
Heart Rate (BPM)	663 ± 42	674 ± 15	696 ± 17	671 ± 45

** *P_Ctrl;Shamvs.Ctrl;TAC_ < 0.01*;

## *P_Ctrl;TACvs.CIP−OE;TAC_ < 0.01*.

Gene expression was detected to confirm the above observation. The expression levels of fetal genes, including Nppa, Nppb, and Acta1, which are often elevated in cardiac hypertrophy, were further increased in METTL5-cKO TAC hearts (Figures 2K–M). Similarly, the expression levels of genes related to fibrosis, such as Col1a1, Col3a1, Fn1, and Postn, were dramatically increased in METTL5-cKO group under stress (Figure 2N). RNA sequencing were further performed to monitor the globe shift of transcriptome. A total of 392 upregulated genes, including Nppa and Nppb, and 146 downregulated genes (|Log_2_ fold change|>1; *p* < 0.05) were detected (Figure 2O and Supplementary Table 5). GSEA analysis showed the activities of several cardiac hypertrophy-related KEGG pathways, such as cardiac muscle tissue development, cell growth, muscle tissue development and tissue remodeling, were enhanced (Figure 2P). Together, these data consistently demonstrated that the loss of METTL5 promoted cardiac remodeling induced by pressure overload.

### METTL5 Mediates Hypertrophic Growth of Cardiomyocytes

**Figure 3:**
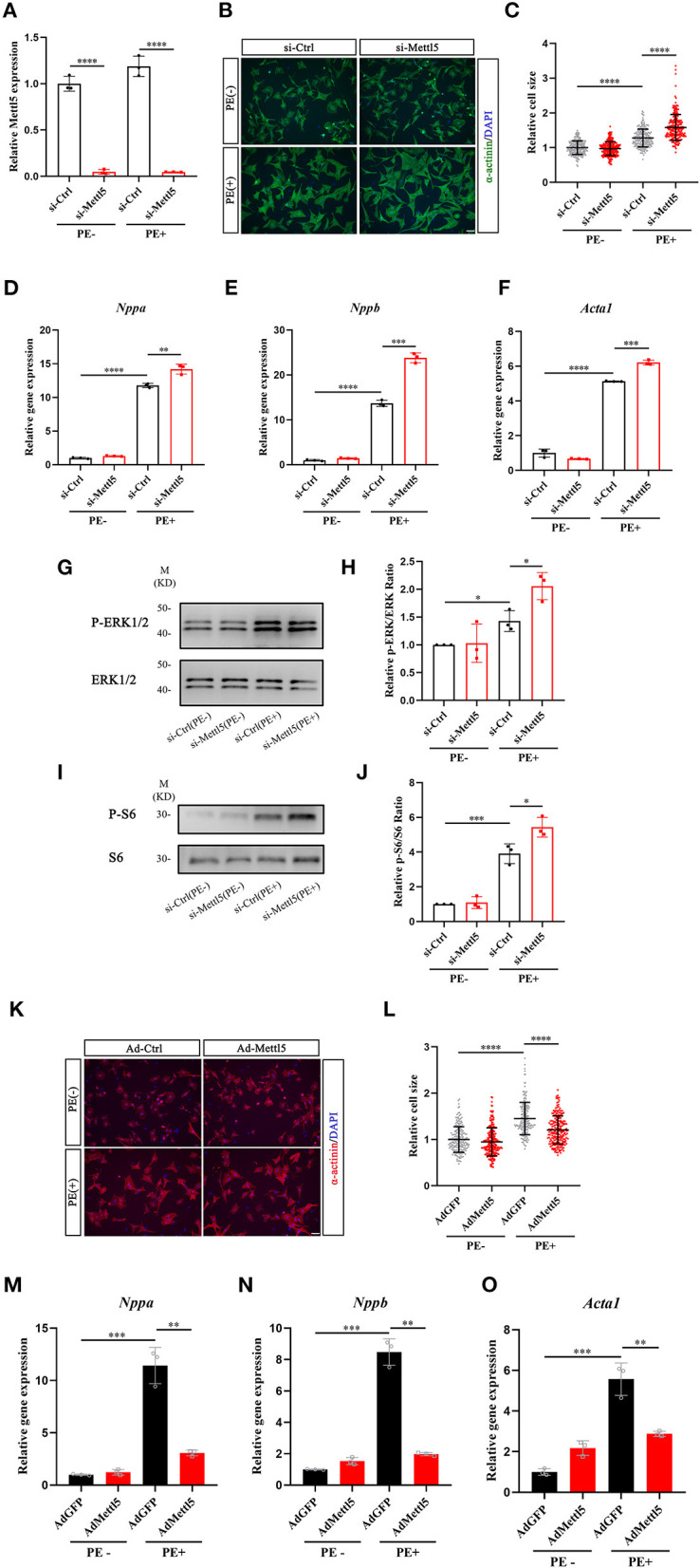
METTL5 promotes hypertrophic growth of cardiomyocytes *in vitro*. **(A)** qRT-PCR detecting the knockdown of METTL5 in neonatal rat ventricular cardiomyocytes (NRVMs) with siRNA. *n* = 3 in each group. **(B)** Representative images of immunostaining of NRVMs transfected with si-METTL5 or control siRNA (si-Ctrl) with or without PE treatment. α-actinin labels cardiomyocytes. DAPI marks nuclei. Scale bar = 50 μm. **(C)** Quantification of the size of cardiomyocytes in panel B. More than 200 cells are measured in each group. **(D–F)** Relative gene expression of hypertrophy markers in NRVMs transfected with si-METTL5 or si-Ctrl with or without PE treatment. *n* = 3 in each group. **(G,H)** Detection of the expression of phosphorylated and total ERK in NRVMs transfected with si-METTL5 or si-Ctrl with or without PE treatment by western blotting. Protein level is quantified and the ratio of phospho-ERK to total ERK is presented. *n* = 3 in each group. **(I,J)** Detection of the expression of phosphorylated and total S6 in NRVMs transfected with si-METTL5 or si-Ctrl with or without PE treatment by western blotting. Protein level is quantified and the ratio of phospho-S6 to total S6 is presented. *n* = 3 in each group. **(K)** Representative images of immunostaining of NRVMs transduced with Ad-METTL5 or control virus (Ad-GFP) with or without PE treatment. α-actinin labels cardiomyocytes. DAPI marks nuclei. Scale bar = 50 μm. **(L)** Quantification of the size of cardiomyocytes in **(K)** More than 200 cells are measured in each group. **(M–O)** Relative gene expression of hypertrophy markers in NRVMs transduced with Ad-METTL5 or Ad-GFP with or without PE treatment. *n* = 3 in each group. The unpaired *T*-test was used for 2-group comparisons. *P < 0.05, **P < 0.01, ***P < 0.001, ****P < 0.0001.

Our data indicated that METTL5 was dominantly expressed in cardiomyocytes. We therefore suggested that METTL5 could regulate hypertrophic growth of cardiomyocytes in a cell-autonomous manner. The expression of METTL5 was knocked down in isolated neonatal rat ventricular cardiomyocytes (NRVM) (Figure 3A). Quantification of cell size indicated that loss-of-function of METTL5 promoted phenylephrine (PE)-induced hypertrophic growth of cardiomyocytes (Figures 3B,C). To confirm this observation, the detection of gene expression showed that knockdown of METTL5 further promoted the expression of hypertrophic marker genes, including Nppa, Nppb, and Acta1, in PE-treated NRVMs (Figures 3D–F). The enhanced levels of phosphorylation of ERK1/2 and S6 ribosomal protein are often connected to cardiac hypertrophy. Western blotting showed that the expression levels of both phosphorylated proteins were further elevated in the PE-treated METTL5 knockdown NRVMs (Figures 3G–J). On the contrary, we asked whether forced expression of METTL5 in NRVMs showed the opposite effect on hypertrophy. Indeed, forced expression of METTL5 with adenovirus repressed PE-induced hypertrophic growth in NRVMs as evident by the smaller cell size of Ad-METTL5-treated NRVMs (Figures 3K,L and Supplementary Figure 4). In addition, forced expression of METTL5 dramatically inhibited the induced expression of Nppa, Nppb, and Acta1 by PE treatment (Figures 3M–O). Collectively, these data demonstrated that METTL5 regulated the hypertrophic growth of cardiomyocytes.

### SUZ12 Is a Key Regulator of METTL5-Medited Cardiac Phenotype

**Figure 4:**
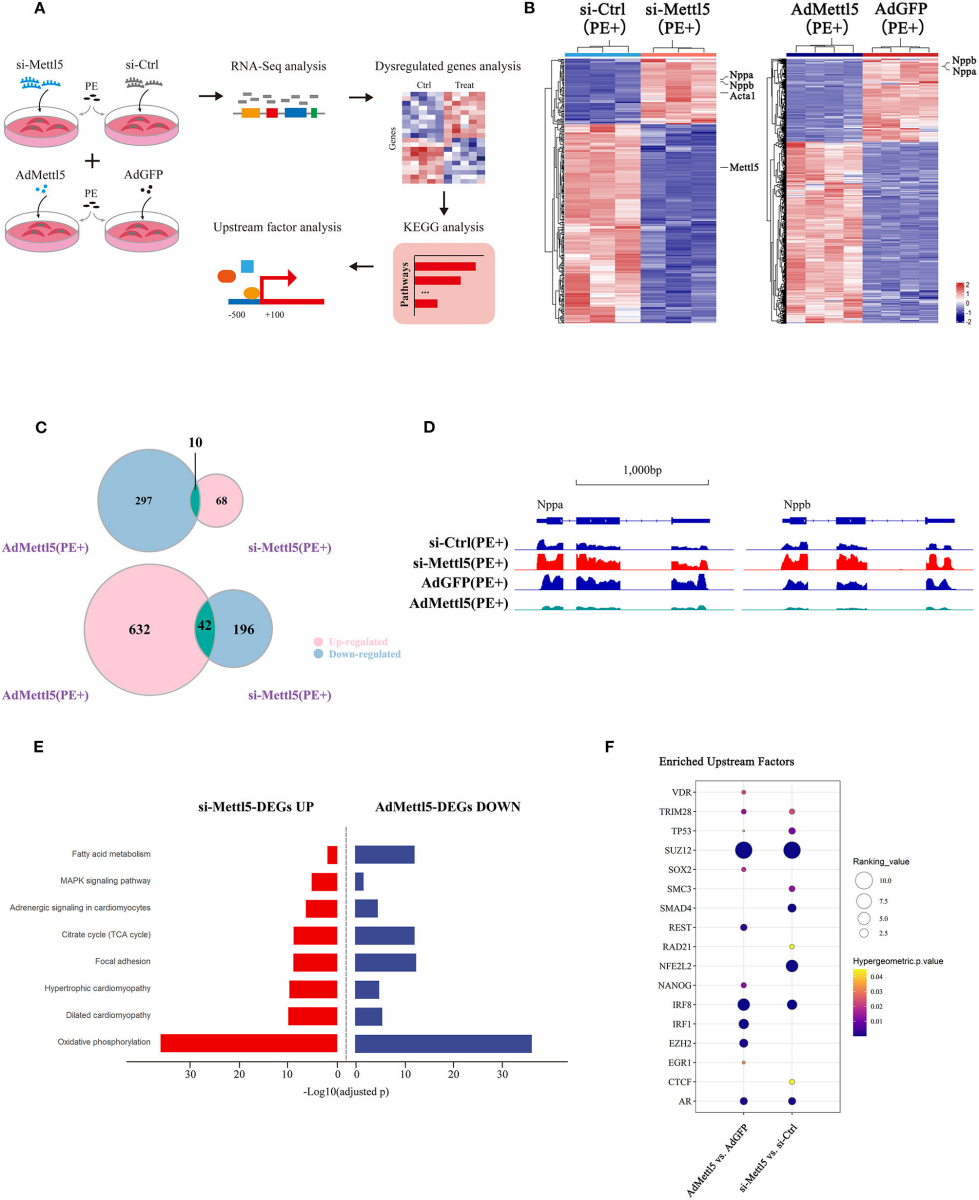
Transcriptome analyses reveal SUZ12 as a key mediator of METTL5-regulated cardiac phenotype. **(A)** Schematic showing the experimental design and workflow of transcriptome analyses of siRNA and adenovirus-treated NRVMs under the stimulation of PE. **(B)** Hierarchical clustering of differentially expressed genes in groups of si-METTL5 vs. si-Ctrl and AdMETTL5 vs. AdGFP under the stimulation of PE. (|Log2FoldChange|>1, adjusted *P*-value < 0.05). **(C)** Venn diagram showing the overlap of upregulated- and downregulated-genes in groups of si-METTL5 vs. si-Ctrl and AdMETTL5 vs. AdGFP under the stimulation of PE, respectively. (|Log2FoldChange|>1, adjusted *P*-value < 0.05). **(D)** The genomic view of reads in RNA-seq for hypertrophic marker gene Nppa, Nppb in the IGV browser. **(E)** KEGG pathway enrichment analysis of dysregulated genes in groups of si-METTL5 vs. si-Ctrl and AdMETTL5 vs. AdGFP under the stimulation of PE (adjusted *P*-value < 0.05). **(F)** Upstream factor analysis by X2K of the top 500 dysregulated genes (|Log2FoldChange|>1, adjusted *P*-value < 0.05; ranking by adjusted *P*-value) in groups of si-METTL5 vs. si-Ctrl and AdMETTL5 vs. AdGFP under the stimulation of PE. The upstream factors were selected by Hypergeometric *p*-value < 0.05. The algorithm of independent hypothesis weighting (IHW) in DEseq2 was used for calculating the adjusted *P*-value of the RNA-seq data.

To explore the molecular mechanism downstream of METTL5 in cardiomyocytes, transcriptomic shift caused by gain- and loss-of-function of METTL5 in NRVMs were monitored by RNA sequencing followed by a series of analyses (Figure 4A). A total of 316 and 981 dysregulated genes (|Log_2_ fold change|>1; *p* < 0.05) were detected in si-METTL5 and Ad-METTL5 treated NRVMs under the stimulation of PE, respectively (Figure 4B and Supplementary Tables 6, 7). Analyses indicated that 52 dysregulated genes, including Nppa, Nppb, and Acta1, were overlapped but showed an opposite expression trend of dysregulation between Ad-METTL5 and si-METTL5 treated NRVMs (Figures 4C,D). Multiple key pathways of cardiac hypertrophy, such as Adrenegic signaling and MAPK signaling, were significantly altered in the KEGG analyses, that showed an opposite direction of alteration in si-METTL5 treated NRVMs vs. Ad-METTL5 treated NRVMs (Figure 4E). We further asked whether these dysregulated genes were controlled by one or more upstream regulators. Dysregulated genes from both datasets were subjected to upstream factor analysis with two independent algorithms, X2K (28) and Lisa (29). Excitingly, SUZ12, a core component of PRC2 complex, was the top hit of the list from both analyses (Figure 4F and Supplementary Figure 5). Given that PRC2 complex has been recently reported to participate in regulating cardiac hypertrophy (30, 31), our data could suggest that SUZ12 was a key upstream regulator mediating the METTL5-related cardiac phenotype observed above.

### METTL5 Translationally Regulates the Expression of SUZ12

**Figure 5:**
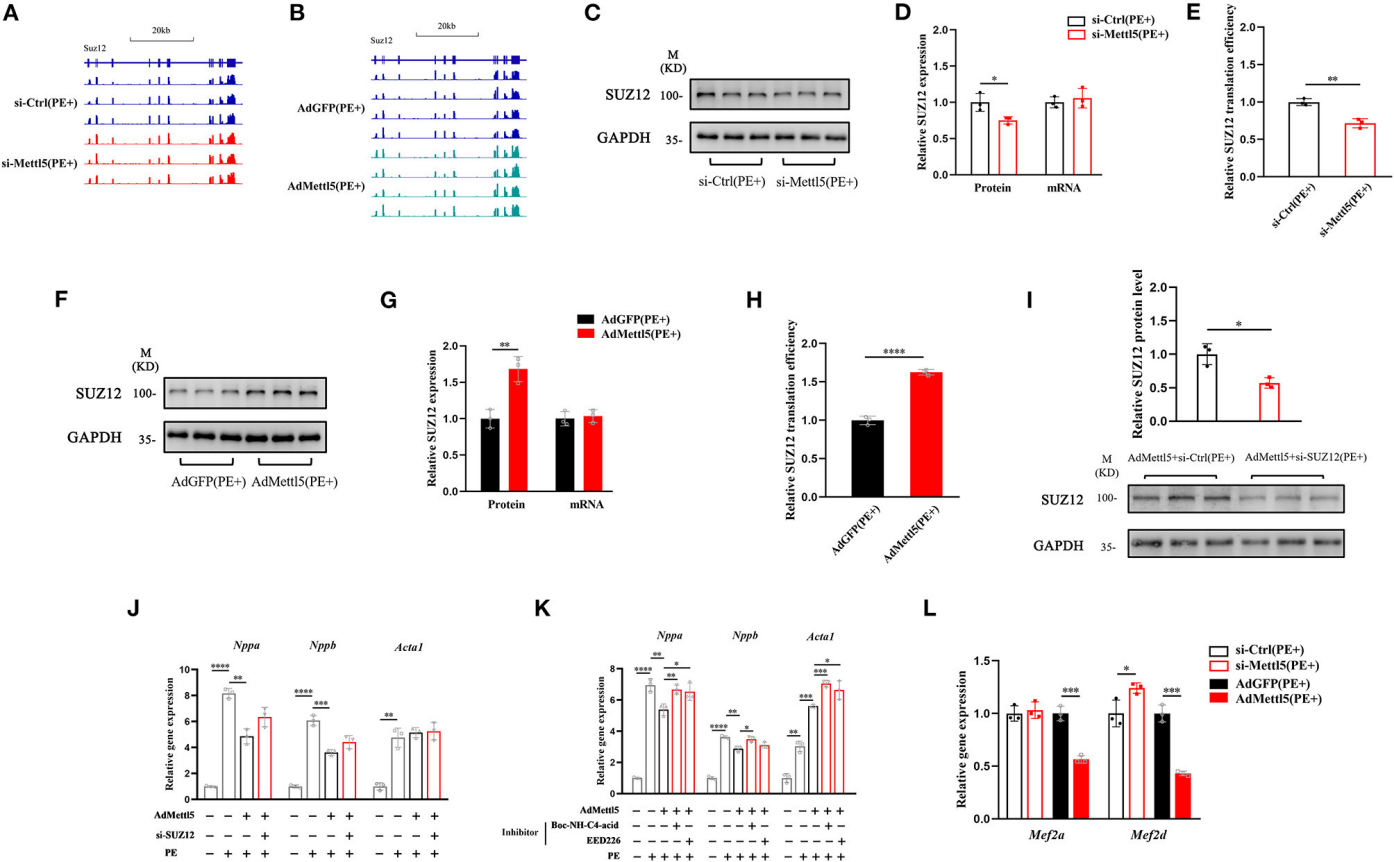
METTL5 regulates the translation efficiency of SUZ12. **(A,B)** The genomic view of SUZ12 mRNA reads of RNA-seq of si-METTL5 vs. si-Ctrl and AdMETTL5 vs. AdGFP under the stimulation of PE in the IGV browser. **(C)** Detection of the expression of SUZ12 in si-METTL5 and si-Ctrl treated NRVCs under the stimulation of PE. GAPDH serves as control. **(D)** Quantification of SUZ12 protein level and qRT-PCR detection of the relative gene expression of SUZ12 in si-METTL5 and si-Ctrl treated NRVCs under the stimulation of PE. **(E)** The translation efficiency (TE) is presented by calculating the ratio between transcript level and protein level. *n* = 3 for each group. **(F)** Detection of the expression of SUZ12 in Ad-METTL5 and Ad-Ctrl treated NRVCs under the stimulation of PE. GAPDH serves as control. **(G)** Quantification of SUZ12 protein level and qRT-PCR detection of the relative gene expression of SUZ12 in Ad-METTL5 and Ad-Ctrl treated NRVCs under the stimulation of PE. **(H)** The translation efficiency (TE) is presented by calculating the ratio between transcript level and protein level. *n* = 3 for each group. **(I)** Detection and quantification of the knockdown of SUZ12 in Ad-METTL5 treated NRVCs under the stimulation of PE. GAPDH serves as control. *n* = 3 for each group. **(J,K)** Relative gene expression of hypertrophy markers in NRVMs treated with (H) si-SUZ12 or (I) SUZ12 inhibitors under Ad-METTL5 or Ad-GFP transfection with or without PE treatment. *n* = 3 in each group. **(L)** Relative gene expression of Mef2a and Mef2d in METTL5 knockdown and overexpression NRVCs and control cells under the stimulation of PE by RT-PCR. *n* = 3 in each group. The unpaired *T*-test was used for 2-group comparisons. *P < 0.05, **P < 0.01, ***P < 0.001, ****P < 0.0001.

Next, we asked how METTL5 regulated the SUZ12-mediated signaling in cardiac hypertrophy. First, we carefully checked the transcript level of SUZ12 from transcriptome analyses of Ad-METTL5 and si-METTL5 treated NRVMs. To our surprise, no significant alteration of SUZ12 expression is found (Figures 5A,B). METTL5 is known to regulate mRNA translation via modifying nucleotide (m^6^A) in 18s rRNA (24, 27). Recently, METTL5-mediated translational control of differentiation regulator FBXW7 has been reported to play a key role in ES cell differentiation (32). Therefore, we suspected that METTL5 regulated the translational efficiency of SUZ12 mRNA in cardiac hypertrophy. Protein and mRNA expression of SUZ12 were detected in si-METTL5 treated NRVMs with PE induction. As a result, protein expression of SUZ12 was significantly decreased with no alteration of mRNA expression (Figures 5C,D), indicating that the translational efficiency of SUZ12 mRNA was repressed when expression of METTL5 was reduced (Figure 5E). On the contrary, gain-of-function of METTL5 promoted the translational efficiency of the SUZ12 mRNA, evidenced with the increased SUZ12 protein level without change in its mRNA expression level (Figures 5F–H). To validate the role of SUZ12 in METTL5-mediated inhibition of cardiac hypertrophy, we performed a rescue experiment by knocking down SUZ12 (Figure 5I). The expression of hypertrophic marker genes Nppa and Nppb, which was repressed by the overexpression of METTL5, was partially rescued by the SUZ12 knockdown (Figure 5J). Furthermore, the partial rescue effect in cardiac hypertrophy was also observed in the treatment of SUZ12/PRC2 inhibitors, EED226 and Boc-NH-C4-acid, during METTL5 overexpression (Figure 5K). Our data indicated that SUZ12 is an important regulator mediating the inhibitory function of METTL5 in cardiac hypertrophy. Mef2a and Mef2d are two major Mef2 family members expressed in the heart and key transcriptional factors for activating hypertrophy-related downstream genes (33, 34). Furthermore, PRC2 complex has been reported to regulate the expression of Mef2 family members (30). Therefore, we detected the expression of Mef2a and Mef2d in NRVMs with PE induction. As indicated, the expression levels of both Mef2a and Mef2d were significantly downregulated in NRVMs with forced expression of METTL5; while that of Mef2d was significantly increased after si-METTL5 treatment (Figure 5L). Taken together, our results suggested that the SUZ12(PRC2)-Mef2d axis could be critical in the regulation of cardiac hypertrophy by METTL5 mainly through modulating the translational efficiency of SUZ12 mRNA.

## Discussion

Nowadays, heart failure is still a deadly disease without effective cure. The complexity of the gene regulatory network underlying the disease development and the lack of fully understood molecular mechanism prevent us from developing novel and effective therapies targeting heart failure. Transcriptional and translational regulation are two critical control steps in the central dogma. Huge amount of efforts have been spent on investigating the transcriptomic shift in the failing heart, giving important insights into key signaling pathways and transcriptional factors in regulating the disease progression. Moreover, the recent findings of m^6^A RNA modification connect the alteration of mRNA translational efficiency to heart failure, indicating the importance of translational control underlying the pathogenesis. However, little is known about how the key step of translation is regulated in the failing heart. In this study, we showed that METTL5 participated in the translational regulation of cardiac remodeling. Forced expression of METTL5 in cardiomyocytes repressed cardiac hypertrophy via modulating the translation of SUZ12 mRNA. In the future, investigation with a model using cardiac-specific overexpression of METTL5 *in vivo* should be carried out to test the therapeutic potential of controlling mRNA translation via METTL5 in delaying cardiac hypertrophy and the progression of heart failure.

METTL5 was ubiquitously detected in mouse organs, which was consistent with the expression data from Genotype-Tissue Expression Project (GTEx) in human. However, it was surprising to find that METTL5 was predominantly expressed in cardiomyocytes. This may suggest that METTL5 could mainly regulate the physiology and/or pathology of the heart. Although the decreased faction of polysome in the METTL5 loss-of-function system has been observed in several reports (24, 27), which indicates the translational efficiency of global mRNAs is affected; however, it seems that it is not entirely true when individual mRNA is evaluated. Ignatova et al. showed that only ~500 transcripts with altered ribosome occupancy without concordant changes in mRNA levels in METTL5-KO ES cells (24), and a portion of these transcripts was even increased in translational efficiency. Therefore, the affected gene target(s) responsible for the observed phenotype could be context-dependent. For example, the decreased translational efficiency of FBXW7 affected by the loss of METTL5 has been reported to be mainly responsible for promoting mouse ES cell differentiation (32). In this study, our data indicated that the enhanced cardiac hypertrophy was mainly mediated by the decreased translation of SUZ12 mRNA in METTL5-depleted cardiomyocytes. More tissues or cell types are warrant to be investigated for the loss-of-function of METTL5 to gain more information of its function of translational regulation in the future.

**Figure 6:**
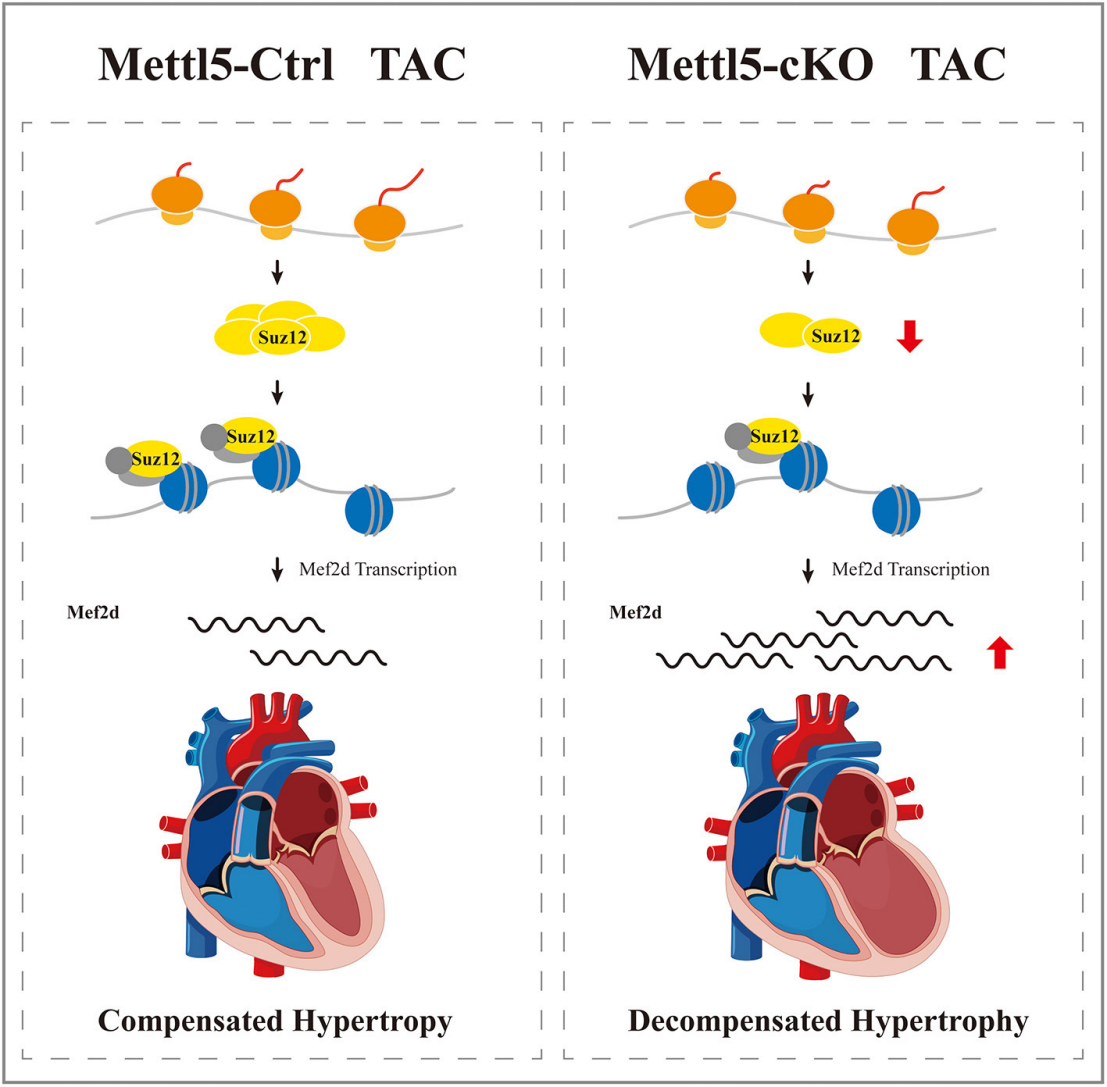
A proposed working model of METTL5 in cardiac remodeling.

In summary, our study uncovered that METTL5 was a key regulator of cardiac hypertrophy, highlighting the importance of regulation at the step of mRNA translation during the development of heart disease. The SUZ12-controlled PRC2-mef2d axis was identified in this study as the main signaling cascade mediating the function of METTL5 in cardiac remodeling (Figure 6). In the future, further investigation should be carried out to dissect the detail mechanism to explain how translational regulation and epigenetic regulation are coupled in the gene regulatory network during the development of cardiac hypertrophy and heart failure.

## Materials and Methods

### Human Samples

Left ventricular (LV) tissues were collected from patients with dilated cardiomyopathy (DCM) during heart transplantation performed in the First Affiliated Hospital, Sun Yat-sen University. In brief, diseased hearts were removed at the time of transplantation and LV tissue was subsequently dissected and snap-frozen. We used LV samples from not implanted healthy hearts to serve as controls (Supplementary Table 1). All the procedures followed the protocol approved by the First Affiliated Hospital, Sun Yat-sen University, Guangzhou, China. All procedures conformed to the 1964 Helsinki declaration and its later amendments or comparable ethical standards.

### Animal Studies

All experiments involving animals in this study were reviewed and approved by the Medical Ethics Committee of The First Affiliated Hospital, SunYet-sen University. Mice were housed under a 12 h light/dark cycle under pathogen-free conditions and with free access to standard mouse chow and tap water. This study conformed to the Guide for the Care and Use of Laboratory Animals published by the US National Institutes of Health (8th Edition, National Research Council, 2011). The METTL5-flox allele mice containing a floxed exon 2–4 of METTL5, which was engineered by CRISPR-mediated homologous recombination, was bred with αMHC-Cre mice (35) to generate cardiomyocyte-specific METTL5 knockout mice. Transverse aortic constriction (TAC) surgery was performed as previously described (36). 2–4% Isoflurane (in oxygen) was used for anesthetization and heating pad was used to maintain the mouse body temperature during the transverse aortic constriction surgery. Anesthesia of the mouse was performed with a nose cone delivering 2–4% Isoflurane in oxygen via small animal ventilator. The setting of ventilator was 110–120 breaths per min with a tidal volume of 0.1 mL under constant monitoring of the inspiratory pressure. At the end of the experiments, mice were killed with the intraperitoneal injection of an overdose of sodium pentobarbital (200 mg/kg).

### Echocardiography Examination

Echocardiographic measurements of mice were performed using a Visual Sonics Vevo 2,100 Imaging System (Visual Sonics, Toronto, Canada) with an 18–38 MHz MicroScan transducer (model MS400). Heart rate and left ventricular (LV) dimensions, including diastolic and systolic wall thicknesses and LV end-diastolic and end-systolic chamber dimensions were measured from two dimensional (2D) short axis under M-mode tracings at the level of the papillary muscle. LV mass and functional parameters such as percentage of fractional shortening (FS%) and ejection fraction (EF%) were calculated using the above primary measurements and accompanying software.

### Histology and Immunostaining

Mouse heart tissues were dissected out, rinsed with PBS, and fixed in 4% paraformaldehyde (pH 8.0) overnight. After dehydration through a series of ethanol baths, samples were embedded in paraffin wax according to standard laboratory procedures. Five micrometer sections were stained with H&E for routine histological examination with light microscope.

For Sirius red/Fast green staining, sections were fixed with pre-warmed Bouin's solution at 55°C for 1 h and then washed in running water. Sections were stained in 0.1% fast green solution for 10 min, then washed with 1% acetic acid for 2 min. After rinsing in tap water, sections were stained in 0.1% Sirius resolution for 30 min. After staining, sections were dehydrated and cleared with xylene. The images were examined with light scope and quantified with Image-Pro Plus software.

To quantify the cross-sectional area of cardiomyocytes, heart sections were deparaffined and stained with Wheat Germ Agglutinin (Alexa Fluor 594 Conjugate WGA; 1:200, Invitrogen) for labeling the cardiomyocyte membrane. Stained sections were examined with a fluorescence microscope (Zeiss; Imager.Z2), and the cross-sectional area of cardiomyocytes in the papillary muscles was quantified with ImageJ software.

### qRT-PCR and Western Blot Analysis

Total RNAs were isolated using Trizol Reagent from cells and tissue samples. For qRT-PCR, 2.0 μg RNA samples were used for cDNA synthesis. In each analysis, 0.1 μL cDNA pool was used for qPCR. The relative expression of interested genes was normalized to the expression of GAPDH, β-actin or 18S. Primers for qRT-PCR were listed in Supplementary Table 8.

For western blotting analyses, tissue samples and cells were lysed in radioimmunoprecipitation assay (RIPA) buffer containing 1 mM PMSF and then denatured at 98°C for 10 min. Samples were subsequently analyzed by SDS-PAGE and transferred to 0.45 μm PVDF membranes. Blocking and blotting with primary antibodies were performed in Tris-buffered saline with Tween-20 (TBST), supplemented with 5 and 3% Bovine Serum Albumin (BSA), respectively. The antibodies used in this study included the following: ant-METTL5 (Proteintech; 16791-1-AP), anti-FLAG M2 (Sigma-Aldrich; F1804), anti-GAPDH (Cell Signaling Technology; cat #2118), anti-β-tubulin (Sigma-Aldrich; T0198), anti-phospho-Erk1/2 (Cell Signaling Technology; cat #4370), anti-total-Erk1/2 (Cell Signaling Technology; cat #4695), anti-phospho-S6 (Cell Signaling Technology; cat #4858), anti-total-S6 (Cell Signaling Technology; cat #2217), anti-SUZ12(Cell Signaling Technology; cat #3737). The membrane was incubated overnight at 4°C with the primary antibodies and washed three times with TBST buffer before adding horseradish peroxidase (HRP)-conjugated secondary antibodies. Specific protein bands were visualized using the Immobilon Western chemiluminescent HRP substrate (Millipore; WBKLS0500) by ImageQuant LAS4000 Mini (GE Healthcare).

### METTL5 Expression Vector and Adenoviral Construction

The human METTL5 coding sequence was cloned into the adenoviral empty vector harboring the murine cytomegalovirus (mCMV). The shuttle vector and an adenoviral backbone plasmid, pAd-ΔE1E3, was then transfected in 293AD cells with PEI (1 mg/mL) to produce packed adenovirus. Subsequently, 293AD cells were transduced with adenovirus for viral amplification. Adenovirus was collected, purified and concentrated by gradient centrifugation with the ViraTrap Adenovirus Purification Miniprep Kit, according to the manufacturer's instructions (Biomiga; V1160).

### Cardiomyocyte Culture

Neonatal rat ventricular cardiomyocytes (NRVCs) were prepared as previously described (37). Briefly, NRVCs were isolated from 1- or 2-day-old neonatal Sprague-Dawley rat hearts by repeated enzymatic dissociation. All isolated cells were pre-plated for 1 h to remove fibroblasts. Non-adherent cells were then plated on 0.5% gelatin-coated plates and cultured in DMEM high-glucose medium (Gibco) containing 10% fetal bovine serum, sodium pyruvate (1×), GlutaMAX (1×) and antibiotics. Cardiomyocytes were changed into serum-free medium after plating for 18 h and transduced with adenovirus for 24 h prior to hypertrophic agent PE (20 μM) treatment. Suz12/PRC2 inhibitors, EED226 (20 μM, HY-101117, MCE), and Boc-NH-C4-acid (50 μM, HY-W014099, MCE) were used in the PE treatment in the rescue experiments. The inhibitors were dissolved in DMSO and manipulated according to the manufacturer's instructions. For the treatment of siRNA, 50 nM siRNA targeting METTL5 or SUZ12 transcript and control siRNA (from GenePharma) was transfected into cardiomyocytes by using Lipofectamine RNAiMAX transfection reagent. 6 h later, media with transfection reagent were removed and cardiomyocytes were then treated with the hypertrophic agent PE (20 μM) by changing the PE-contained serum-free medium. Cells were harvested 24 h after PE treatment for RNA isolation or 48 h after PE treatment for immunochemistry and total proteins or 0.5 h for detection of phosphorylated protein.

### Immunofluorescence Staining

Cardiomyocytes were washed with PBS three times and fixed with 4% PFA for 15 min. After washing with PBS, cells were then blocked with 3% BSA in PBS containing 0.1% TritonX-100 for 1 h, followed by incubation with anti-sarcomeric a-actinin (1:200; Abcam; ab9465) in blocking buffer for 2 h at room temperature. After three times wash with PBS, cells were incubated with anti-mouse secondary antibodies conjugated with Alexa 488 (1:1,000; Thermo Fisher Scientific; A-11029) or Alexa 594 (1:1,000; Thermo Fisher Scientific; A-11032), together with the nuclear stain DAPI (0.1 mg/mL; Sigma; D9542) in PBS for 1 h. Images ware captured using a fluorescence microscope (Olympus; IX71). The surface area of cardiomyocytes was quantified with ImageJ software.

### RNA-Seq Data Analysis

Raw reads were aligned to the rat or mouse genome (Rattus_norvegicus.Rnor_6.0; Mus_musculus.GRCm38) using HISAT2 (38). We generated read counts using StringTie (v2.1.4) with default options (39). Differentially expressed gene was calculated using DESeq2 (40). To view the distribution of reads, the bam files were converted to bigwig format using deepTools (v.3.3.1) (41) and visualized with the Integrative Genomics Viewer (IGV).

The raw data of RNA-seq in this study were deposited in Gene Expression Omnibus (GEO) database of the National Center for Biotechnology Information (NCBI) (Accession: GSE186615).

### Upstream Factor Analysis

The dysregulated genes in si-METTL5 (vs. si-Ctrl) and AdMETTL5 (vs. AdGFP) were analyzed with the parameter of |Log2FoldChange|>1 and adjusted *P*-value < 0.05. Two independent algorithms, X2K (7, https://maayanlab.cloud/X2K/) and Lisa (8, http://lisa.cistrome.org/), were used for upstream factor analysis, and top 500 dysregulated genes (ranking by adjusted *P*-value) were selected and subjected to analysis according to the instructions.

### Statistics

Values are reported as mean ± SD unless indicated otherwise. For multiple group comparisons, a *post-hoc* Tukey's test was performed when ANOVA reached significance. In addition, the unpaired *T*-test was used for two-group comparisons. *P*-values < 0.05 were considered statistically significant.

## Data Availability Statement

The datasets presented in this study can be found in online repositories. The names of the repository/repositories and accession number(s) can be found in the article/Supplementary Material.

## Ethics Statement

The studies involving human participants were reviewed and approved by the Medical Ethics Committee of The First Affiliated Hospital, SunYet-sen University. The patients/participants provided their written informed consent to participate in this study. The animal study was reviewed and approved by the Medical Ethics Committee of The First Affiliated Hospital, SunYet-sen University. Written informed consent was obtained from the owners for the participation of their animals in this study.

## Author Contributions

Z-PH conceived the project, designed and analyzed the experiments, and wrote the manuscript. YH, TD, and SG performed most of the experiments. XZ, YC, CL, SLi, XL, and YD contributed to human sample acquisition and western blotting analysis. LW, TL, TX, and GD generated METTL5-cKO mice, performed transverse aortic constriction surgery, and collected mouse heart samples. YH and TD contributed to the echocardiographic data acquisition and analysis. TD contributed to bioinformatic analyses of RNA-seq data. KL, DZ, XT, and SLin supervised the data analyses and reviewed the manuscript.

## Funding

This work was supported by the National Natural Science Foundation of China (81873463 to Z-PH, 81970429 to DZ, and 81900350 to SLi), the Guangdong Basic and Applied Basic Research Foundation (2019B151502003 to Z-PH, 2019A1515011956 to DZ, and 2018A030313448 and 2021A1515010433 to YC), and the donation for scientific research from the Terry Fox Foundation to Z-PH.

## Conflict of Interest

The authors declare that the research was conducted in the absence of any commercial or financial relationships that could be construed as a potential conflict of interest.

## Publisher's Note

All claims expressed in this article are solely those of the authors and do not necessarily represent those of their affiliated organizations, or those of the publisher, the editors and the reviewers. Any product that may be evaluated in this article, or claim that may be made by its manufacturer, is not guaranteed or endorsed by the publisher.
