# Supplementary material for: Loss of m6A Methyltransferase METTL5 Promotes Cardiac Hypertrophy Through Epitranscriptomic Control of SUZ12 Expression
Source: Front Cardiovasc Med. 2022 Feb 28;9:852775. doi: 10.3389/fcvm.2022.852775 (PMC8920042; doi:10.3389/fcvm.2022.852775)
Supplement: Supplementary file 1 [file Data_Sheet_1.pdf]

Supplemental Table 1. Information of controls and dilated cardiomyopathy (DCM) patients

	Age	EF（Teich）
**Control 1**	44	N/A
**Control 2**	50	N/A
**Control 3**	35	N/A
**DCM 1**	44	15
**DCM 2**	55	20
**DCM 3**	53	29
**DCM 4**	24	24
**DCM 5**	41	30
**DCM 6**	48	51

Supplemental Table 2. Dysregulated genes in 1-month-old METTL5-KO mice and their control littermates (|Log2 fold change|>1; adjusted p value<0.05)

gene	log2FoldChange	padj
Pde4b	1.512515926	1.52E-05
Kcne1	1.056882247	0.017082
Bc1	-1.036390277	1.79E-05
Bace2	-1.191149812	0.008529
Camkk2	-1.81701219	4.30E-14
Gck	-2.447987665	0.027809

Supplemental Table 3. Echocardiography examination of 12-week-old METTL5-cKO mice and their control littermates at baseline

	Ctrl (N=6)	METTL5-cKO (N=5)
**IVS;d (mm)**	0.771±0.041	0.771±0.045
**IVS;s (mm)**	1.553±0.126	1.515±0.082
**LVID;d (mm)**	3.729±0.128	3.757±0.158
**LVID;s (mm)**	1.809±0.129	1.824±0.122
**LVPW;d (mm)**	0.751±0.037	0.780±0.050
**LVPW;s (mm)**	1.462±0.058	1.524±0.052
**EF (%)**	83.34±2.02	83.31±1.58
**FS (%)**	51.52±2.28	51.48±1.76
**LV Mass (mg)**	97.92±4.58	102.16±14.04
**LV Mass (Corrected, mg)**	78.34±3.66	81.73±11.23
**LV Vol;d (uL)**	59.32±4.96	60.41±6.12
**LV Vol;s (uL)**	9.93±1.82	10.12±1.68
**Heart Rate (BPM)**	695±28	677±19

Note: no significant difference is found between groups.

Supplemental Table 4. Dysregulated genes in 12-week-old METTL5-cKO mice and their control littermates (|Log2 fold change|>1; adjusted P value<0.05)

gene	log2FoldChange	padj
1700094D03Rik	8.032108985	2.24E-10
Eprn	1.732204199	5.45E-03
Ndnf	1.685995503	1.48E-10
Hif3a	1.551898411	1.04E-02
Ucp2	1.499359359	1.31E-17
2310015A16Rik	1.348429423	2.25E-04
Ccn4	1.336924556	2.43E-02
Phlda3	1.329296199	3.28E-14
Mt2	1.225053535	1.31E-02
Cenpf	1.191593424	2.76E-02
Slc38a1	1.105510767	3.87E-07
Nppa	1.101695885	2.59E-05
Lad1	1.097417598	7.45E-03
Plekha7	1.070312992	8.58E-03
Gm12167	-1.009987155	4.72E-02
Egln3	-1.021029684	7.73E-03
Gm49891	-1.030029095	5.97E-03
Fam220a	-1.030898896	1.46E-02
Kcnv2	-1.059635743	6.64E-05
Gm50114	-1.095484964	2.76E-02
Nav3	-1.144316093	0.034911963
Poln	-1.336456918	0.040837925
Mylf-ps	-1.601774307	0.000842769
Myl1	-1.669069147	3.28E-14
Pcdhgb5	-1.859201619	0.0000651
Pcdhgc4	-1.970000709	0.047152681
Aldob	-2.782888662	1.92E-18
Eps8l1	-3.3573309	0.02577082

Supplemental Table 5. Dysregulated genes in hearts of METTL5-cKO mice and their control littermates 4 weeks post TAC (|Log2FoldChange|>1, adjusted P value< 0.05)

gene	log2FoldChange	padj
Gm20716	11.97213329	0.001271079
Kctd12	10.69486237	0.003018025
Gm49808	8.13523307	7.39E-09
Cthrc1	8.084430935	1.65E-08
Itih2	7.460849808	2.90E-09
Ecrg4	6.955680497	0.029256696
Tnni1	6.761214164	5.41E-06
Ctsw	6.453869734	2.55E-06
Gm2174	5.953757573	0.033613235
Sprr1a	5.539698997	4.85E-07
Atp6v0a4	5.508878009	0.003457597
Cfb	4.909251972	4.13E-13
Comp	4.631249324	4.22E-06
Col9a2	4.597344061	5.36E-06
Serpinb9b	4.477072809	0.001521354
Ltbp2	4.450594873	1.45E-07
Krt18	4.440405034	0.008169512
Crlf1	4.438598848	0.000545818
Chil1	4.410706612	0.024610851
Slc13a4	4.361389922	4.04E-06
Frem1	4.357639953	5.39E-05
Ereg	4.218292046	8.85E-06
Thbs4	4.153562288	2.74E-07
Col12a1	4.138504363	2.97E-12
Fam180a	4.054294242	2.20E-06
Gpr39	3.979881619	0.00124914
Gm11382	3.976208708	0.000128831
Gdf15	3.933705933	1.76E-08
Col8a2	3.795898989	2.99E-05
Serpinb1c	3.763297512	2.92E-11
Postn	3.661261986	4.65E-07
Fmod	3.633674672	3.21E-08
Cilp	3.602439898	4.47E-05
Nppa	3.507561387	1.90E-32
Serpina3n	3.343630621	5.44E-10
Plcxd2	3.332168865	0.006465715
4833412C05Rik	3.26225422	0.003006241
Adamts20	3.256340022	0.032686459
Dio2	3.184946542	2.62E-06
Nox4	3.117584902	1.46E-06
Dclk3	3.104138801	0.007937139
Gm13054	3.090597179	7.42E-17
Anxa8	3.089644053	5.39E-05
Gdf6	3.009624137	1.60E-07
Lox	2.982820915	1.33E-06
Lrp8	2.948636243	1.05E-06
Timp1	2.946348515	7.44E-11
Ngef	2.921343028	0.00031077
Serinc2	2.905345183	0.02201473
Sfrp2	2.901242772	6.26E-10
Ccn5	2.855473577	2.19E-06
Clca3a2	2.81777142	0.009716661

Nrip3	2.810465733	0.001965817
Nmrk2	2.80776127	0.000421528
Adamts8	2.753043397	5.75E-05
Cxcr6	2.698268298	1.13E-05
Dkk3	2.682042321	4.27E-09
Ccn2	2.66790453	4.29E-10
Pamr1	2.631907387	4.68E-06
Gm30873	2.611381383	0.0001258
Mfap4	2.583020612	2.93E-06
Frzb	2.576061376	2.61E-10
Tent5b	2.557758885	1.31E-16
Col8a1	2.519031024	4.28E-05
Kif1a	2.516721244	1.64E-05
St14	2.510805221	0.00246735
Spp1	2.476045143	0.000182623
Prob1	2.474029556	0.00076474
Gm15079	2.433217287	0.000822601
Gins2	2.43128877	0.005401248
Syndig1	2.42252796	0.020457615
Ankrd2	2.381630749	1.17E-11
Egr2	2.375069066	4.49E-08
Grip1	2.363498286	0.002870594
Star	2.353800326	2.20E-06
Ptx3	2.324827511	8.09E-06
Svep1	2.290105965	1.97E-05
Psca	2.261564625	0.013202836
Eppk1	2.251492914	0.000779177
Pak3	2.2324842	0.006011201
Itgbl1	2.19081826	4.49E-08
Fibin	2.125574137	0.000136446
Tubb3	2.106898172	8.94E-05
Ryr3	2.094971358	0.026492654
Mfap5	2.094902329	6.44E-06
Il7r	2.091960492	0.013509048
Fgl2	2.084816735	7.32E-13
Cd300lf	2.084053837	0.032262174
Pou3f1	2.07288221	4.62E-06
Adamts4	2.037467247	0.000421528
Rgs11	2.028255944	0.001782595
Sfrp1	2.017524441	0.001005498
Xirp2	2.000635806	3.97E-05
Lman1l	1.997235453	9.71E-16
Panx1	1.993625561	3.47E-06
Col5a2	1.986809962	0.000374699
Slc1a3	1.984705117	4.18E-05
2200002D01Rik	1.969842954	1.15E-05
Rab15	1.951256821	0.001801862
Tgfb2	1.946800709	5.08E-08
Vcan	1.931083129	1.29E-05
Angptl7	1.930878945	4.19E-08
Eprn	1.917624383	0.000498903
Synpo2l	1.915587304	6.73E-13
Nupr1	1.909616703	1.40E-05
Clec11a	1.909319548	9.03E-05
Gask1a	1.907118868	0.000471688
Col3a1	1.907017958	0.000188942
Fbn1	1.88747106	0.001633446
Col14a1	1.886156607	1.47E-06

Phlda3	1.884785031	5.48E-25
Gm21451	1.883578992	0.001108876
Pcdhgb1	1.882246802	0.02440316
Gpr35	1.876899557	0.004193284
Serpinf1	1.873863513	7.40E-05
Kcnj14	1.870212965	0.020771529
Sv2b	1.868342621	0.035137183
Slc9a3	1.862788465	0.000338275
Adamtsl2	1.861389846	4.03E-13
Clca3a1	1.859717313	0.00012756
Stk32b	1.845450884	0.001805779
Pcdhga6	1.841498851	0.016753449
Efhd2	1.838280832	2.14E-31
Ankrd1	1.825635724	7.35E-14
Edn3	1.818166021	2.09E-05
Thbs1	1.777776516	9.50E-07
Uck2	1.769062293	1.76E-08
Fhad1	1.76838064	0.038440329
Pdgfrl	1.764585807	0.000152038
Aldh1a3	1.756959232	0.000135823
Il2rb	1.754649026	0.025876387
Tbx15	1.749091676	0.000712642
C4b	1.744102318	2.85E-07
Gm29773	1.742838549	0.019053091
Ankrd23	1.740993182	0.000328423
Htra4	1.734398232	0.000250556
Col1a1	1.730719716	0.000776526
Apod	1.727952847	1.65E-08
Tmem45a	1.727294473	0.000688481
Bgn	1.719299425	4.64E-08
Rbp1	1.716173421	9.73E-06
Klhl29	1.715188032	0.029193901
Mthfd2	1.704765957	8.28E-08
Dusp4	1.700987115	0.023764214
Tnc	1.697617562	0.001005498
Gm17228	1.694061843	2.58E-12
Enpp1	1.685000738	0.000180959
Loxl2	1.680617392	1.09E-05
Serpine1	1.677688903	2.22E-11
Col1a2	1.675110187	5.88E-07
Snap91	1.673152032	8.74E-06
Zfp365	1.672531106	0.000822069
B4galnt1	1.671975671	0.001198003
Cda	1.668364933	0.000605513
Fstl1	1.666526973	0.000128831
Omd	1.657972756	0.000740368
C4a	1.648811678	2.16E-05
Adam12	1.639760862	0.013042189
Acta1	1.63707264	3.58E-21
Rnf208	1.636441478	0.04314375
Fbxl2	1.6357036	0.005656118
Fbn2	1.619443145	0.041436941
Adamts2	1.584535096	4.06E-06
Fstl3	1.580833319	2.31E-11
Col16a1	1.57502111	1.70E-05
Hbegf	1.571760779	1.68E-08
Pacsin1	1.566542705	0.016259793
E230013L22Rik	1.559068137	0.00035131

Nlrc3	1.558332743	0.0001393
Orai2	1.549899667	0.00025072
Clu	1.544883958	7.61E-07
Havcr2	1.544584039	0.017734444
Gm12295	1.532235801	0.002920535
Eln	1.528949003	0.011011077
Krt80	1.514158267	0.000101963
Bend4	1.513522044	0.001975385
Uchl1	1.512967597	2.85E-07
Ssc5d	1.506165242	5.52E-05
Ccdc136	1.504135877	5.88E-07
Ppm1e	1.503449206	2.22E-06
Nppb	1.490190817	2.14E-06
Nt5e	1.489519817	0.00025072
Lingo1	1.487133601	0.030010994
Rasgef1a	1.468881355	0.012745227
Vgll3	1.462933285	0.000247747
Col4a3	1.46093999	0.001135758
Lgals3	1.459400457	0.000147805
Rcan1	1.458133564	2.92E-11
Rnd1	1.454202039	0.009323053
Sphk1	1.444882305	0.000828579
Tcf7	1.443822017	0.040892233
Meox1	1.443704038	8.28E-05
Rrm2	1.443386514	0.045429775
Dpysl3	1.435506143	1.36E-07
Tlr8	1.434655483	0.003777271
Lcn2	1.431038703	3.48E-07
Itih5	1.430062642	2.95E-05
Gm16284	1.429217342	0.009680281
Ackr4	1.425892578	1.57E-07
Mmp23	1.420086719	8.75E-08
Fgf6	1.417520217	9.16E-05
Ydjc	1.414397566	0.028563476
Vsig4	1.402028263	0.005013156
Syt12	1.401988216	1.47E-06
Sec1	1.401264426	0.01047994
Lbp	1.401027167	1.64E-05
Shisa3	1.397028226	0.012270983
Scara3	1.395041491	0.000756838
Pcbd1	1.39444122	0.009682047
Ism1	1.392524417	0.015445645
Sema3d	1.391113748	0.003181723
Ndnf	1.390999361	1.18E-07
Serpina3c	1.388303817	0.039557408
Ddias	1.382574296	0.045581545
Myc	1.382064047	1.59E-05
Tenm3	1.379783548	0.00138278
Loxl3	1.378575789	0.003523317
Fbxo17	1.377691144	0.021968132
Mgp	1.377253602	2.61E-10
Cgref1	1.376244199	0.019871964
Nabp1	1.360216854	0.000183631
Dbn1	1.358966895	2.98E-07
Prkab2	1.35209265	2.43E-06
Trf	1.351368254	2.62E-06
Rtn4	1.351365933	1.63E-07
Nr4a1	1.3369132	2.92E-05

Csf2rb2	1.335628905	0.014511421
Spc25	1.331692604	0.018909167
Cx3cl1	1.330208701	2.58E-05
Tmem119	1.329173101	0.00012781
Ahnak2	1.327229334	8.28E-05
Emp1	1.325155822	1.63E-05
Fmn1	1.321777117	0.035958555
Met	1.320675692	0.016296558
Fcrls	1.318534168	0.000525293
Gm47692	1.317981649	9.32E-13
Lad1	1.316819416	0.002711263
Gxylt2	1.313105369	0.000628009
Etv4	1.311124118	0.031304273
Igsf23	1.309109152	0.001946888
Comtd1	1.308531418	5.54E-06
Ptgs2	1.305893882	0.000183631
Prelp	1.304735345	3.07E-05
Loxl1	1.304208525	3.70E-05
Pi16	1.304076223	1.72E-11
Klrg2	1.303643363	0.04242595
Gm30085	1.300023075	0.023708469
Rasl11b	1.298858482	4.82E-09
Mybpc2	1.293383288	4.28E-21
Baalc	1.293011091	0.028652839
Gli2	1.29272059	0.008648864
Npl	1.291307165	0.020241607
Ildr2	1.287364258	0.0130571
Gm16332	1.281660284	0.000179898
Srpx2	1.274651687	0.002380076
Pwwp3b	1.273562977	0.004630532
Ncam1	1.272608466	0.000102197
Paqr6	1.266932776	0.026466739
Gm13481	1.264504764	0.003072406
Ctsk	1.263981	1.48E-05
Serpinb6b	1.262691896	8.36E-09
Fgf14	1.2626482	0.004880143
C230062I16Rik	1.260526817	0.00211421
Gpx3	1.259355669	0.01823673
Fbln5	1.247269445	0.000292497
Sntb1	1.245430268	3.36E-05
Mt2	1.243731754	6.69E-06
Ccr2	1.243443828	0.025121489
Egr3	1.234495535	0.000671358
Clec4n	1.231981855	0.002776524
Nkd2	1.231256229	0.031366093
Plscr1	1.22728462	0.005741142
Scn1b	1.22683339	5.05E-09
Abhd15	1.225689727	0.029711804
Mdfi	1.225191046	0.044609047
Pcdh9	1.220415474	0.022981509
Sulf1	1.220046903	2.07E-05
Chaf1a	1.215349951	0.000349324
Trem2	1.208152785	0.005953077
Pcolce	1.205753784	3.36E-05
Col5a1	1.204568142	8.94E-05
Rhod	1.204454434	0.000195029
Ahsg	1.203446337	1.48E-05
Fzd2	1.203442653	2.80E-05

Kif26b	1.202147319	0.003111284
Clmp	1.198167738	0.003031769
Ddah1	1.196788862	0.021158263
C1qtnf6	1.196752826	1.70E-05
1500009L16Rik	1.195090992	3.70E-05
Zfp697	1.195016996	6.65E-07
Igfbp7	1.192200727	0.000262886
Cdkl2	1.192167184	1.73E-06
Arfip1	1.189401428	0.007163204
E530011L22Rik	1.188830964	0.003128127
Ass1	1.188067304	0.001188056
Ace	1.186331735	1.43E-07
Aff3	1.184874298	0.011930279
Apcdd1	1.184723747	0.001428842
Il17re	1.184106251	0.020685467
Rab31	1.174480471	7.91E-11
Fn1	1.174374927	1.30E-05
Grk5	1.174141388	1.60E-07
Fndc1	1.171875827	0.001050276
Blk	1.170612041	0.003018106
Thsd7a	1.170580008	1.41E-05
Fat1	1.168516704	2.87E-06
Srpx	1.167577106	0.004678766
Itga11	1.167542515	0.001951401
Map1a	1.166966158	2.35E-05
Inhbb	1.166515492	0.009147908
Slc22a4	1.165014596	0.003280885
Arhgdig	1.162956283	0.013156017
Shisa4	1.161234535	2.47E-07
Tpbg	1.160578036	0.043479979
Sh3rf3	1.159311933	0.035998815
Aqp8	1.155731662	0.019590386
Mllt11	1.146788644	0.000485124
Ccn4	1.145792703	0.00719019
P3h2	1.133941208	0.00443777
Gpc6	1.132181548	0.006315625
Pdpn	1.131532468	0.001078012
Gask1b	1.127985769	0.004405607
Slc38a4	1.127907121	2.63E-07
Antxr1	1.127570458	0.000186001
2310015A16Rik	1.12689878	0.000367386
Srgap3	1.122165009	0.048293691
Pcsk5	1.118589074	0.009591772
Nbl1	1.113985576	0.000248495
Cnksr1	1.113063687	5.72E-07
Ms4a6c	1.112214678	1.55E-06
Gm12840	1.108033224	0.027642594
Plcg2	1.104726118	2.40E-06
Plekha4	1.104458573	0.000607602
Gm4788	1.101909092	0.000395173
F2rl1	1.100883707	0.024865182
Cfh	1.100373914	1.86E-05
Gabra3	1.100305613	0.011147651
Olfml2b	1.098202354	0.000792331
Pak6	1.097563892	7.28E-07
Cfhr2	1.094772862	0.004088447
Amot	1.092934363	0.005955052
Gm2395	1.090774619	0.023956029

Slc38a1	1.090564829	0.000125953
F2r	1.088587818	2.71E-06
Camkk1	1.088571848	0.005499719
Eya2	1.088217944	0.00297577
Gria3	1.087389414	0.007527736
Dok1	1.086378918	0.022426252
Tdrkh	1.085067898	0.002631167
Gm2412	1.084190486	0.028304435
Slc41a2	1.084156892	0.041975228
Prss23	1.079414555	9.72E-06
Fzd1	1.078797569	2.09E-05
Rasd1	1.073051932	0.036791362
Pqlc3	1.070367074	0.001367131
Cytip	1.069136671	0.029181507
Pfkp	1.061715131	8.70E-07
Adam9	1.058820142	0.000234747
Bcl2	1.058311716	0.000848835
Ankrd45	1.056834504	0.001185851
Ccn1	1.056421375	1.68E-06
Hectd2os	1.054274244	6.92E-08
Lrp1	1.052394989	2.99E-09
Sypl2	1.046373857	0.004719181
Maff	1.045665086	0.000792142
Ccdc80	1.045357838	3.56E-09
Ptgis	1.039706515	0.000240386
Dap	1.037517342	0.000617638
Gpnmb	1.037266051	0.005207011
Atp10a	1.035522637	0.008888008
Ptrh1	1.033286651	0.039557408
Slc1a4	1.033165554	0.00388024
Des	1.03255181	1.89E-08
Fam122b	1.031126075	0.022548353
Scx	1.030013082	0.00107206
Fhl1	1.026037527	5.66E-05
Slco2a1	1.025543614	0.007195471
Pam	1.019448575	5.52E-05
Epn3	1.019005579	1.04E-17
Serpine2	1.018424424	0.005931587
Fbln2	1.017712541	2.09E-05
AI506816	1.015404873	8.11E-08
Gm3604	1.014308356	0.042686866
Csf2rb	1.013615666	0.044191
Stc2	1.012605383	0.003025282
Ccdc68	1.008729607	0.037609822
Pard6b	1.008474057	0.000338275
Spta1	1.008237817	0.001778542
Pdlim2	1.007515393	0.001672916
Snhg15	1.007042234	0.00951044
Lhfpl2	1.005104234	0.014185789
Mmp2	1.000973694	2.35E-05
Tgtp1	-1.005703688	0.043381993
Tppp	-1.008929799	0.000913595
Gm45012	-1.009437879	0.007995341
Pdp2	-1.010145605	2.18E-06
Sema6c	-1.011312601	0.000359114
Slc40a1	-1.011375171	0.00036105
Rassf9	-1.013815813	0.00026482
Rpl3l	-1.014840013	0.000181983

Enpp2	-1.022890204	0.045612302
Mx2	-1.02530805	0.025431256
Gbp5	-1.027782108	3.68E-10
Gm8979	-1.029133061	8.17E-05
Gm49891	-1.036642456	0.001483948
9030612E09Rik	-1.037606614	0.018087081
Gm36372	-1.043254277	0.000689687
Ybx2	-1.04531494	0.005174258
Gm31013	-1.04897939	2.22E-06
Ifi44	-1.055295345	2.36E-05
Pdzd9	-1.06019607	0.047057028
Aplnr	-1.060245979	0.000462079
Wnk2	-1.069125282	0.004899758
Cmss1	-1.069971518	0.000219354
Cox7a1	-1.077535552	8.11E-08
Tnfsf10	-1.08034434	5.20E-08
Gm8989	-1.080791535	0.000144995
Amy1	-1.0836877	0.000467303
Il2ra	-1.091153265	0.018302455
Rassf10	-1.091307981	0.032854174
Mamdc4	-1.094829872	0.003025282
4930481A15Rik	-1.095462331	0.001521354
Inka2	-1.095608698	0.001801752
Egflam	-1.105042433	0.000248749
Cpeb3	-1.110257894	1.12E-06
Slc26a6	-1.111319972	0.000190272
Gm826	-1.114646159	0.001105376
Cryba4	-1.117489994	0.048661991
Pla2g4e	-1.117867203	0.028634452
Tmem150c	-1.11875175	2.93E-06
Ech1	-1.120753804	4.44E-06
Gm37691	-1.123877127	0.044469007
Gm13479	-1.12588897	0.042771241
Cmpk2	-1.129148327	4.43E-07
Gm47644	-1.139074346	4.03E-10
Grip2	-1.14645566	0.009488232
Mhrt	-1.147494523	0.001043954
Cacna1s	-1.148774195	2.96E-07
Ppl	-1.162355636	0.011930279
Aqp7	-1.162725198	0.00049062
Misp3	-1.164877199	0.003970605
Gm43361	-1.165435663	0.008463582
AU020206	-1.178901421	8.69E-08
Ret	-1.178910594	0.001908066
D830026I12Rik	-1.184482968	0.004644871
Gm49477	-1.18547487	0.007385543
Cdh23	-1.186341876	0.000127115
Fam131a	-1.186963177	0.016495376
Epb41l4b	-1.188708648	0.039557408
Egf	-1.188848122	0.002370196
BB218582	-1.190308609	0.00013128
9630028I04Rik	-1.192343362	0.03097062
Gal3st3	-1.194745781	0.000268336
Dqx1	-1.195806542	0.03856469
Slc22a3	-1.205610765	0.00169109
Tmem82	-1.210455985	1.55E-06
Gfra3	-1.228048164	0.010364379
Rsad2	-1.259614901	2.22E-11

Gfra4	-1.261751231	0.007163204
Gm43359	-1.264872181	0.016952342
Sox10	-1.266338024	0.029711818
Rbfox1	-1.270230633	0.005005897
Kcnd2	-1.277988958	0.000130453
Gm45552	-1.278183184	0.02428542
mt-Tq	-1.286327314	4.83E-05
Slc22a1	-1.289364581	0.015053379
mt-Ti	-1.304221219	7.18E-05
Frmpd3	-1.305265762	0.007918909
Pitpnm3	-1.307024835	0.006668702
Smco1	-1.307707483	0.000288531
Ttll1	-1.321458473	7.97E-06
Gm38077	-1.32424806	0.001630007
Alox12	-1.326753111	0.0024574
Immp2l	-1.332275156	1.19E-16
Lrrc4b	-1.349876998	0.001514817
H3c14	-1.355618394	0.005062085
Slc2a12	-1.356514208	5.89E-05
Gal3st2c	-1.359687339	0.010831393
Gm31251	-1.35983729	9.90E-14
Efemp1	-1.364432185	0.002869476
Gm4956	-1.367107065	0.029570134
Odf3b	-1.409757368	0.048708581
Adamts7	-1.434402204	3.25E-09
Ctla4	-1.440860731	6.28E-05
Slc29a4	-1.442932413	0.042023069
Mas1	-1.447561563	0.039083064
Lgals4	-1.495856071	0.010789858
Rhbdl3	-1.504313228	1.51E-05
Mir208b	-1.548150512	4.54E-05
Gm29686	-1.553033211	0.004703081
Phkg1	-1.557272141	5.12E-07
Mme	-1.56587889	0.007824778
Gm50194	-1.566355914	0.00141723
Gm12167	-1.582046514	0.000163922
Gm10435	-1.59367476	0.000195029
Kcnj3	-1.613858296	4.16E-15
Gpr22	-1.614165635	0.000180959
Gm32369	-1.621457966	0.018074039
Pcdhgb6	-1.625588034	0.005577148
2310039L15Rik	-1.648197262	0.000568552
Klhl33	-1.649881696	0.000182623
Ccl11	-1.658497564	0.030033369
Gm19277	-1.660830131	0.008463582
4732487G21Rik	-1.66398311	0.004696123
Adcy1	-1.669141316	4.77E-05
Gm24474	-1.680880921	0.001269405
Penk	-1.68730687	0.049428146
Gm42971	-1.692386897	0.008438242
Gm20683	-1.692544919	0.016259793
9430062P05Rik	-1.748982666	0.004129331
Etfbl	-1.76061202	0.046859484
Strit1	-1.829584168	9.70E-07
Poln	-1.88663443	0.005335153
Olfr1396	-1.891458001	8.06E-05
Gm48607	-1.935668917	0.035329455
Uckl1os	-1.99507199	0.023448886

Syn3	-2.032499011	0.030389952
Ces1d	-2.04989205	0.027309859
Fbp2	-2.096803303	3.56E-13
Sbk2	-2.160766474	4.70E-05
Cngb3	-2.253063719	4.18E-05
Pfkfb1	-2.261691955	7.37E-08
Kcnv2	-2.32466176	8.11E-08
Aqp4	-2.426221886	0.000981612
Gm45909	-2.483456359	0.027398877
Clcn1	-2.610232272	5.17E-12
Glcci1	-2.922303357	0.000290926
Gm10635	-3.029929232	0.00068555
Cyp1a1	-3.107545331	0.005678401
Fxyd3	-3.255868872	0.001322389
Gm37240	-3.258028641	0.000115427
Acsm5	-3.396048975	0.022308912
Gm44503	-3.500736839	0.004335364
Lypd2	-3.913823848	2.16E-05
Mfrp	-4.177372021	0.00473729
Wif1	-4.740597872	3.41E-06
Aldob	-4.922233379	3.79E-07
Gm20547	-7.027802621	3.98E-08

Supplemental Table 6. Dysregulated genes in si-METTL5 (vs. si-Ctrl) treated NRVMs under PE stimulation (|Log2FoldChange|>1, adjusted P value< 0.05)

gene_name	log2FoldChange	padj
AABR07053509.2	7.251885001	2.35E-07
Cldn3	3.00609331	6.84E-12
Nwd2	2.39924066	5.23E-07
Hs3st2	2.285240621	7.13E-05
Gdf15	2.024110952	9.38E-23
Plcxd2	1.980088805	0.000235058
Emcn	1.936210982	0.027756319
Aqp7	1.928846988	1.19E-07
Areg	1.906943831	2.44E-13
Sez6l	1.886583243	5.03E-30
Drd1	1.846964899	0.036755163
Gprc5a	1.700503954	7.18E-50
Atp12a	1.665393771	0.0271543
Slc38a3	1.637460108	5.45E-09
Phf24	1.605471633	8.58E-47
Plvap	1.598445783	4.71E-06
Anxa8	1.565814914	0.009366384
AC119762.6	1.505691916	1.66E-05
Slc20a2	1.500712897	2.72E-204
Nppb	1.457730385	1.84E-143
Atcay	1.448610963	1.74E-21
LOC680227	1.424609492	0.003132919
Bdnf	1.409529011	1.93E-31
Adm2	1.400674526	6.46E-06
Atf3	1.359050311	1.48E-20
Nr4a3	1.334588614	1.04E-16
Cavin4	1.298644029	2.41E-39
Xirp2	1.295724206	3.42E-18
Tbkbp1	1.288729356	3.30E-39
Rcan1	1.277891115	6.40E-114
Ildr2	1.275154195	7.09E-17
Pnoc	1.270617773	5.43E-16
Pxdc1	1.245297475	4.62E-43
Nppa	1.244791318	2.80E-65
Syt12	1.242580596	6.38E-16
Bdkrb2	1.241449352	1.89E-16
Ptchd1	1.224369452	1.17E-06
Rnf39	1.223679159	3.24E-06
B3gat1	1.218901395	0.010481548
Stmn4	1.218521418	0.028782218
Cyp1a1	1.213640783	0.001479287
Syde1	1.211606183	5.26E-81
Fhl1	1.195891975	7.97E-72
Dusp26	1.167536579	0.011452002
Myct1	1.160627219	4.44E-05
Pianp	1.155972125	9.45E-12
Rapgef4	1.152480934	3.68E-10
Tmeff1	1.149858415	6.00E-56
Prg4	1.142646665	1.47E-09
Isg20	1.138099911	7.26E-09

F2rl1	1.1335561	9.68E-25
Ano3	1.125184619	2.20E-15
Eda2r	1.12387114	2.18E-20
Acta1	1.120687608	3.34E-18
Lgmn	1.111692018	7.25E-98
AABR07008030.1	1.090803206	0.044933258
Dusp10	1.087247348	3.28E-14
Rasd1	1.081153659	3.91E-06
Irf6	1.076011995	1.10E-30
Meox1	1.072639249	2.57E-07
Casq1	1.068746876	1.87E-97
Gadd45g	1.05965591	6.00E-56
LOC292543	1.059600518	6.89E-19
Nfkbiz	1.059048132	0.032544913
Kcna6	1.058669306	4.71E-06
Abcb9	1.042571468	0.005082943
Ckmt1	1.04019286	1.43E-06
Plin4	1.039826417	2.72E-09
Fam167a	1.037401234	6.72E-05
Lmod3	1.035288432	0.000584065
Uap1	1.035169945	2.75E-57
Mfsd4a	1.033512737	0.016972413
Asb9	1.027799989	5.45E-07
Bmp2	1.014494753	9.87E-24
AABR07030598.1	1.013392799	0.007511447
Fgf16	1.005280358	5.54E-19
Frmd5	1.004200988	4.99E-15
Ift43	1.002503824	2.45E-33
Cd200r1	-1.000923608	3.87E-19
Ephb1	-1.001249246	0.028276322
Pole2	-1.002115712	0.000241325
Smoc2	-1.002384673	3.60E-06
Igdcc4	-1.002498599	4.95E-10
Mcf2l	-1.005113684	0.010136534
Map2k6	-1.005473385	0.001237891
Arrdc4	-1.005755442	2.41E-36
Ahr	-1.009435308	4.50E-34
Sfrp2	-1.011339433	2.03E-07
Kank4	-1.011909832	0.009262125
Gbp2	-1.015521642	1.16E-05
Tnfrsf1b	-1.015773296	4.22E-11
Dtx3l	-1.018667225	1.33E-13
Socs3	-1.020864483	3.25E-12
Ssc5d	-1.021380247	5.09E-35
Cd302	-1.022164531	4.02E-18
LOC103689947	-1.023743919	2.59E-11
Pla2g2a	-1.024412504	0.014484753
Adamts5	-1.024816134	2.19E-26
Tmem45a	-1.028932488	5.81E-07
Psmb8	-1.030422968	2.15E-05
Plpp1	-1.030809269	5.96E-68
Fcgr1a	-1.034560012	0.007047572
Srpx	-1.043624281	9.56E-26
RT1-CE3	-1.046519021	8.33E-08
Slit3	-1.046568613	2.70E-64
Cdh3	-1.049093176	4.13E-25
Igsf10	-1.051445506	3.83E-33
Col23a1	-1.053227529	0.013863276

Nhs	-1.053447346	1.68E-08
Epor	-1.054805393	0.040312381
RGD1560455	-1.054985825	4.61E-06
Inhbb	-1.064344496	1.91E-09
C7	-1.065427151	1.09E-21
Ccdc80	-1.065936855	2.41E-71
Slc16a14	-1.066302245	8.15E-11
Rgcc	-1.068484125	9.62E-05
Lrp2	-1.068717362	0.012275168
Cp	-1.069174087	3.61E-28
Mboat1	-1.070665197	0.001008346
Cyp7b1	-1.071192401	1.19E-07
Sncg	-1.072235083	0.039925332
Ptn	-1.076095472	1.72E-06
Ca13	-1.076432552	4.86E-09
Cdkn1c	-1.080125928	5.56E-15
Bmp6	-1.080833782	8.25E-31
Slfn5	-1.081787979	1.01E-70
Ptx3	-1.082814063	2.47E-24
Il17b	-1.083738345	0.014044209
Ccl12	-1.087812416	6.15E-05
Mrgprf	-1.088113593	6.30E-16
Alpk1	-1.094916134	4.06E-33
Hapln1	-1.095638586	0.017849293
Antxr1	-1.098472385	3.51E-58
Kif24	-1.098802038	0.004194159
Enpp2	-1.099218681	0.009102881
Tac3	-1.107534225	3.60E-05
Ece2	-1.107732301	0.001213481
Commd10	-1.107932582	5.85E-27
Vipr2	-1.108396898	0.008105482
Ly6e	-1.111197374	7.64E-24
Nexmif	-1.115897437	0.013549732
Syt13	-1.117726704	0.013047614
Dtx4	-1.118942658	2.94E-12
Rbfox1	-1.121782696	0.000213015
Gpr88	-1.121932567	1.96E-20
Matn4	-1.122809568	0.014999754
LOC103693323	-1.124898579	1.02E-06
AABR07068316.1	-1.127306974	4.91E-14
Tmem45al	-1.128495721	4.37E-07
Pwwp3b	-1.132375539	4.62E-16
Asic1	-1.141289707	7.57E-24
Bmp3	-1.14236057	1.27E-18
Pcdh19	-1.144151072	4.62E-20
Crlf1	-1.146767322	4.56E-56
Fmo4	-1.150980118	0.000437639
Gprin3	-1.155241631	0.009066472
Prr22	-1.156876662	0.005395632
Nrk	-1.160375784	2.60E-15
Steap4	-1.163130984	0.000378955
Gas7	-1.171946942	6.32E-46
Helz2	-1.172545723	1.60E-14
Slc9a5	-1.173935133	0.008498332
Hgf	-1.176250737	8.01E-20
Bnc2	-1.176855223	0.017213772
Tmem176a	-1.177626822	1.89E-16
LOC691083	-1.177720152	0.000419395

Cpz	-1.179586927	2.82E-19
Nnat	-1.182345548	1.62E-18
AABR07006310.1	-1.185700828	0.005587343
Tlr5	-1.187184939	4.35E-14
Tnfaip6	-1.188197651	9.57E-07
Lfng	-1.19479601	0.000385859
Bmper	-1.195690394	3.33E-09
Pvalb	-1.199132142	0.00063153
AABR07011698.1	-1.201307156	0.000134016
Zc3h12b	-1.202171265	0.044068855
Mmp16	-1.203126218	4.65E-24
LOC24906	-1.211517615	0.000558428
Nnmt	-1.217652179	1.02E-05
Fgl2	-1.220071031	8.21E-29
Ermap	-1.22137022	0.001878009
Cfb	-1.223592142	0.015441067
Rassf4	-1.22841046	7.02E-45
Gbp6	-1.235238841	4.76E-17
Ifi27l2b	-1.236125986	2.43E-05
Mnda	-1.236403415	1.55E-42
AABR07032338.1	-1.242099145	0.007788422
Lypd1	-1.246963518	7.74E-36
AABR07044322.1	-1.248391324	0.002478329
Sorcs1	-1.25373907	6.49E-11
Fmod	-1.257483078	2.55E-62
Cpxm2	-1.260912319	8.39E-12
Jag1	-1.28263446	1.64E-43
LOC103690116	-1.284295069	0.013450566
Mamstr	-1.286871459	0.017262211
Sfrp4	-1.288522913	1.15E-10
Upk3b	-1.295134872	5.03E-07
Cd4	-1.302999932	0.005543744
Adamts3	-1.308971158	1.77E-07
AABR07025140.1	-1.317258862	1.12E-07
Serpinb9	-1.322214259	1.09E-64
Myo7a	-1.330033853	1.44E-24
Them5	-1.340314395	0.000145123
Fbln1	-1.341466114	4.14E-28
Igf2	-1.352753473	3.34E-13
Slc1a1	-1.354546442	3.21E-07
Nkd1	-1.354813153	4.74E-17
Gda	-1.355643797	2.58E-22
Oasl2	-1.357019583	1.32E-18
Dlk1	-1.360139172	0.016782816
Rtp4	-1.362899588	1.56E-08
Vdr	-1.365860272	2.02E-10
Ephb6	-1.366866294	1.11E-19
Cpxm1	-1.384621826	1.10E-07
Lum	-1.385206012	1.02E-11
Mx2	-1.389482981	0.024610283
C1qtnf1	-1.397150897	9.60E-52
Mx1	-1.401635736	1.30E-10
Olfml1	-1.405330833	4.47E-29
RT1-T24-3	-1.411421611	2.21E-09
Uba7	-1.411888053	0.000147981
Drp2	-1.413755158	0.001411717
Clec3b	-1.415035392	5.93E-18
Siglec10	-1.419213215	1.54E-05

Wt1	-1.419311517	1.33E-13
Gpr39	-1.426669912	0.007894622
Dpp4	-1.426729597	1.11E-07
Adam12	-1.428950084	1.61E-52
Efcab1	-1.436811727	0.027186837
AABR07030366.1	-1.438533053	0.001633292
C4a	-1.438823414	4.00E-08
Islr2	-1.452401483	0.044384172
Arhgef19	-1.457704621	1.87E-10
Car2	-1.475527451	5.82E-17
Xaf1	-1.479214892	0.000745433
Aldh1a3	-1.480071367	1.30E-12
Tlr7	-1.480388993	0.012990348
Fgfr3	-1.480975484	5.94E-21
Vsnl1	-1.482661712	5.31E-06
Psmb9	-1.486633582	9.49E-10
Tbxa2r	-1.492555038	3.22E-10
C4b	-1.501332087	2.18E-08
Ifi47	-1.528886592	1.24E-08
Tmem178a	-1.529388059	6.02E-17
Itgb6	-1.531106878	9.65E-07
Adamts15	-1.534582206	9.98E-23
LOC103689965	-1.534630103	2.17E-10
Lrrn4	-1.536569839	2.96E-16
Cfh	-1.538176912	0.039823218
Bmx	-1.538308487	1.51E-06
Mir675	-1.543392754	0.009548464
Dhrs9	-1.581931978	0.007023561
Agtr1a	-1.600751113	8.14E-11
Hpx	-1.616241671	2.51E-10
Gbp1	-1.619529858	0.029084601
Tmeff2	-1.62002525	1.10E-05
Pappa1	-1.629212518	2.73E-06
Grem2	-1.631672228	4.21E-06
Lilrb4	-1.635978754	6.47E-16
Lrrc17	-1.638212427	1.14E-26
Rspo1	-1.640826569	4.81E-06
Igf1	-1.653476018	8.89E-154
C3	-1.663864499	0.000236214
Gbp4	-1.669665858	0.000309095
Wnk3	-1.700039392	9.74E-08
Igfbp3	-1.713238347	1.40E-137
Bst2	-1.724718863	0.003331141
Wdr86	-1.729108393	0.000713077
MGC108823	-1.737510655	1.57E-12
Il33	-1.74851234	1.16E-66
Abi3bp	-1.750205184	1.28E-20
Trpv6	-1.754233351	0.003677962
Vcam1	-1.773773905	8.83E-16
Atp8b4	-1.776557048	0.00013951
Tnn	-1.789464878	1.59E-14
Angpt1	-1.807992647	2.01E-29
Pdgfra	-1.835271696	3.60E-48
Cacna1d	-1.835333799	0.020816906
Scn7a	-1.840936639	8.12E-09
Sult5a1	-1.841999362	0.009300858
LOC100910979	-1.8430017	2.59E-11
Cemip	-1.894411307	2.48E-30

Vegfd	-1.943893144	1.39E-124
LOC102555392	-1.978377594	7.17E-06
LOC100910934	-1.991499578	3.71E-15
Akr1c14	-2.002306532	8.44E-14
Crb2	-2.012501152	0.0061502
Cxcl12	-2.033254969	1.96E-13
Igfbp2	-2.04946359	4.04E-06
RGD1309362	-2.075275118	1.96E-32
Gata3	-2.103330208	3.48E-10
LOC100911545	-2.150778859	6.42E-44
Hpgd	-2.155165358	0.035310804
Mettl5	-2.157490101	2.38E-42
A2m	-2.160792201	6.42E-44
Efemp1	-2.162931251	2.22E-19
Bco2	-2.168047841	0.022246142
Calhm5	-2.172651245	0.012254848
Cilp	-2.175133538	5.43E-37
Reg1a	-2.215547208	2.27E-05
Wnt4	-2.384066355	4.35E-16
C6	-2.603833836	2.48E-30
Mfrp	-2.681419072	0.021583311
Fmo2	-2.829114815	0.032116481
Penk	-2.889278864	2.69E-160
Casp1	-2.909402856	0.010433754
Epyc	-3.067529323	2.30E-06
Hmgcs2	-3.103626207	0.00154996
Cxcl13	-3.495209029	1.22E-42
Igflr1	-3.49784862	9.87E-05
Ca3	-4.31030304	9.38E-64
Slc6a1	-4.416951786	7.35E-16
Oas2	-6.978720443	1.36E-06
Htr3a	-7.050969461	6.84E-07
Pde9a	-7.195418069	2.65E-07
Alox15	-7.403576249	6.21074E-08

Supplemental Table 7. Dysregulated genes in AdMETTL5 (vs. AdGFP) treated NRVMs under PE stimulation (|Log2FoldChange|>1, adjusted P value< 0.05)

gene	log2FoldChange	padj
Bpifb4	8.475125637	4.49E-15
Alx1	8.143455391	1.27E-13
Oas2	7.9671835	7.43E-13
Slc10a4	7.762815165	2.56E-12
AABR07025272.1	7.735171181	3.20E-12
Gabrb2	7.660491414	6.10E-12
Fndc3c1	7.413500891	5.64E-11
AABR07057765.1	7.277211616	1.85E-10
AABR07040695.1	7.11770137	4.28E-10
Fezf1	7.10700069	1.16E-09
Vgll2	7.074133022	1.28E-09
Ccl5	6.916917254	0.008570231
Zbp1	6.87828454	4.22E-09
Slc5a11	6.875707877	2.63E-09
Slit1	6.794513591	0.008278189
Eomes	6.614018295	2.23E-08
Mak	6.424990718	1.60E-07
Grm2	6.35297988	3.51E-07
4933403O08Rik	6.288829473	3.19E-07
Klrk1	6.270176668	7.33E-07
Batf2	6.265065706	3.84E-07
Pcsk4	6.238338787	4.98E-07
Aim2	6.176983426	2.92E-06
Tmprss2	6.066508081	8.64E-18
LOC100362814	5.886944384	0.000590393
AABR07013843.1	5.85047705	2.02E-05
Fbxo39	5.841967356	8.24E-06
Hoxd13	5.837116199	1.78E-05
Ooep	5.720538676	2.32E-05
AABR07040839.1	5.715564555	0.001003337
Txk	5.697628036	0.001082671
Arx	5.636608949	3.54E-05
Rp1	5.590745719	5.35E-05
Pou4f1	5.538631175	0.000114796
Eqtn	5.534882326	0.002146038
St6galnac5	5.518762263	1.38E-11
AABR07036336.2	5.493500279	0.000134733
Igdcc3	5.467015976	0.003014472
Mia	5.40450839	0.000312103
Dok6	5.328551057	0.033035665
Chl1	5.301633766	0.033970536
Scin	5.289093755	0.034819101
Rasgrf1	5.247938672	0.038208651
Dnah7	5.230548789	0.039888592
Mx2	5.212543264	2.69E-20
Foxi3	5.195796402	0.008306728
Serpinb3a	5.193668958	0.008178439
Poln	5.189686082	0.043559324
Sncb	5.181427191	0.000968267
Pacsin1	5.179601947	0.008738592
Kcnc2	5.178552583	0.008752594

AABR07005667.1	5.159707361	0.046427198
Kcnv1	5.131358795	0.010371116
Pitx1	5.125651718	0.001576321
Ca4	5.11997605	0.010392166
AABR07051376.2	5.102316498	0.010952806
RGD1560394	5.049483061	0.013568429
AABR07012302.1	4.996296254	0.003171019
Vnn3	4.971138063	0.017726931
Pcdh8	4.929127415	0.021696871
Rln3	4.9161166	0.004751808
LOC685849	4.914618726	0.002893167
AABR07051426.1	4.908595262	0.021192927
Tekt3	4.905552345	0.022910657
Csmd1	4.902038784	3.50E-13
Cbln1	4.860679404	0.029691418
Tssk4	4.859252782	0.027234101
Gsc	4.851921787	0.028651028
Hoxd9	4.843934839	0.026866024
Brinp3	4.837069177	0.006010831
Pou3f3	4.781100497	2.10E-08
Coch	4.750668	0.034711529
Oas1a	4.742224919	4.41E-08
Tnfsf4	4.682564753	0.010128727
Npc1l1	4.671158844	0.010954528
Rsad2	4.622923488	1.04E-17
AC109891.1	4.594233222	0.021196961
Tacstd2	4.554772692	6.66E-13
Zfp804a	4.471320823	0.001135003
Ifi44l	4.470450915	0.000340493
Gdf7	4.403409393	0.030633713
Isg15	4.296524534	1.52E-34
Fscb	4.291935724	0.045324988
Rgs20	4.291657992	2.66E-09
Htr4	4.266669282	1.08E-22
Runx3	4.263088311	7.72E-05
Myb	4.241386872	0.000479094
Cntn1	4.241135896	0.000115451
Tmprss11d	4.220044069	5.58E-07
Irf7	4.212571225	2.37E-42
Rtp4	4.183023253	8.08E-90
Hoxc6	3.993256672	0.00089483
Usp18	3.983037034	1.97E-14
RGD1305184	3.982067085	5.23E-06
Oasl	3.954282322	1.17E-17
Mx1	3.940034956	4.09E-33
Oas1f	3.938282385	1.18E-12
Nell2	3.925997126	7.97E-120
Oas1g	3.899585243	3.11E-19
Oas1i	3.882135589	2.34E-19
Spock1	3.865304169	2.42E-08
Sbk3	3.856412311	0.001680185
Ifit3	3.85396158	1.28E-17
Sp9	3.849562595	0.00958635
Sptssb	3.843691234	0.002146038
Rec8	3.826931574	0.032349172
Ifi47	3.740856234	1.45E-24
AABR07067520.1	3.729743335	1.61E-15
Bst2	3.724840789	3.82E-41

AABR07059875.2	3.694784429	0.000413293
AABR07018078.1	3.690040146	1.56E-33
Dhx58	3.687680239	6.45E-44
Adcy1	3.68747428	3.98E-10
Elovl2	3.682409069	0.000625143
Chst8	3.649547634	0.00059833
Pax9	3.641857114	0.005052434
Kif17	3.637039547	0.017398048
Ifit1	3.630489562	5.35E-26
Shisa8	3.625958264	3.97E-15
Hsh2d	3.582262799	1.51E-10
Pi15	3.580152238	2.92E-08
Zglp1	3.576082264	0.036633772
Tp73	3.572094131	7.46E-11
Hs6st3	3.545080589	2.87E-13
Slc24a2	3.53107844	4.23E-07
Mdga2	3.51776803	2.48E-32
Oasl2	3.498527899	5.47E-31
Samd9	3.493364739	2.70E-40
Cdh8	3.480194442	1.89E-17
Uba7	3.458126749	1.19E-42
LOC100910979	3.453808558	9.90E-41
Elmod1	3.407570772	2.06E-19
Kcng1	3.38033063	3.41E-07
MGC108823	3.379442834	1.15E-46
Foxg1	3.366726257	0.006068734
Herc6	3.357385037	3.02E-37
Nlrc5	3.348617338	1.46E-05
AABR07025140.1	3.339337312	1.30E-58
AABR07029417.1	3.334340517	8.67E-10
Baat	3.271279995	0.00640037
Crtac1	3.252017855	2.06E-39
LOC102555392	3.213054855	1.02E-17
Gabra1	3.203708907	1.23E-27
Cxcl10	3.166334091	8.70E-19
Akr7a3	3.146684694	3.58E-36
Stox1	3.117738573	0.000485827
Npy1r	3.105863387	8.90E-38
Cmpk2	3.098436341	4.75E-36
Irgm	3.092197551	2.48E-118
Sybu	3.076333665	0.010254054
Esrp1	3.07499941	0.003971085
Isl1	3.050914017	0.019742203
Apol9a	3.045387228	1.20E-22
Macc1	3.03491079	0.047166928
Car7	3.026083569	0.008278189
Npbwr1	3.022986761	0.049807543
Ddx58	3.014183673	1.79E-32
Bpifb1	3.008728476	0.00248361
AC126641.1	2.999824965	1.39E-34
Ifi44	2.986563854	2.27E-21
LOC690507	2.970900669	8.29E-34
Foxd1	2.957634557	2.38E-09
Lrrtm4	2.935434692	3.53E-11
Fam184b	2.897569176	0.010810701
Gbp1	2.89254745	9.70E-55
Mesp2	2.863025963	0.001145458
Igtp	2.845048911	1.03E-45

AABR07052588.1	2.80158607	0.019952868
Xaf1	2.799788475	1.73E-37
U6	2.794108767	0.015626427
Spta1	2.787703551	7.26E-08
RT1-T24-3	2.771454219	1.58E-24
Sp6	2.737908684	0.001524792
AABR07005775.1	2.731966656	0.026382611
LRRTM1	2.710501038	0.000241231
Dtx3l	2.702037244	1.41E-89
AABR07031489.1	2.684378151	9.64E-05
Kcnj4	2.656659351	7.95E-08
Ptger3	2.639858109	1.10E-64
Helz2	2.623791366	4.53E-73
Mapk4	2.620277658	7.28E-13
Iqsec3	2.619484478	2.30E-13
Dab1	2.618344803	2.36E-09
Foxa2	2.60022239	1.76E-06
Parp9	2.594733807	1.20E-54
RGD1309362	2.583398171	1.76E-65
Ifih1	2.582335596	4.75E-33
Dock3	2.56928089	0.005222163
Ano5	2.567083092	0.008349662
AABR07066693.1	2.566519891	0.040792233
Gbp5	2.549008169	1.59E-59
Testin	2.539486688	1.97E-08
LOC100910934	2.530039529	4.71E-32
Parp14	2.527525607	1.00E-146
Mrvi1	2.500601753	2.47E-122
Glp2r	2.48413927	0.046035967
Nrxn1	2.474604389	0.003913049
Nexmif	2.445831588	2.81E-47
AABR07049695.2	2.442040692	0.001768951
MGC105567	2.441332313	4.47E-27
Parp12	2.425682937	9.05E-101
Ddit4l2	2.415250154	6.28E-34
Ctnnd2	2.4139967	1.08E-21
Vwde	2.40031988	0.003288096
Kitlg	2.391804395	3.96E-159
Lix1	2.383414175	6.75E-05
Fam81a	2.340871579	7.10E-96
Lgals3bp	2.337154975	4.12E-109
Rasd1	2.328288332	1.52E-75
Havcr2	2.32661724	0.034855991
Plk5	2.282943793	1.31E-21
Cnr1	2.267590545	3.75E-10
Tbx15	2.253312178	4.20E-08
Kcna1	2.247899239	0.016861217
Nrcam	2.239340567	1.35E-39
Tdrd1	2.238372428	0.014973444
Il6	2.231949968	0.005185471
Msh5	2.207006499	0.005914778
AABR07044322.1	2.191855787	1.40E-11
AABR07059159.1	2.190811876	2.42E-06
Nefl	2.189770372	1.60E-22
Map4k2	2.187752782	3.55E-83
Rnf213	2.176869382	2.46E-93
Dcx	2.167994189	8.83E-05
Tafa2	2.164867534	9.83E-12

Synpr	2.158703837	2.97E-08
AABR07044420.2	2.151269355	4.54E-12
Ina	2.144422083	5.92E-49
Serpini1	2.133571887	5.30E-25
St8sia1	2.131258277	0.000353054
Atg9b	2.131182574	0.015981178
AABR07030091.1	2.124711944	0.017571441
Ifit2	2.121784597	1.09E-104
Rspo3	2.119636981	3.45E-35
Slfn4	2.107904208	1.30E-92
Dlx1	2.10632203	0.01695523
Glp1r	2.10484345	7.38E-09
Tap1	2.095817049	3.06E-79
Tmco5b	2.090044427	0.020707077
Dlx5	2.074738538	5.82E-13
Ugt8	2.06288251	0.020386042
Maob	2.054801033	7.07E-12
RT1-S2	2.036627098	1.37E-10
C1ql1	2.035904907	2.39E-19
Mgat5b	2.034384677	0.033797757
Fam221a	2.017082708	1.85E-27
AABR07067080.1	2.01595339	0.000487021
Gbp4	2.003468693	1.13E-36
Rtp3	1.996834095	1.56E-05
Pgbd5	1.989397505	6.79E-07
AABR07058479.1	1.986716524	0.008518045
Pcdh10	1.983688144	3.48E-12
Kcna7	1.983155532	5.35E-12
Psmb9	1.966888693	3.38E-46
AABR07021804.1	1.95907955	1.03E-08
AABR07044364.1	1.944758013	3.56E-33
Nat8l	1.944276494	2.06E-14
Fbxo2	1.940790988	1.11E-05
Nr5a2	1.929990395	0.007176494
Acsl6	1.922029722	2.22E-07
Hlf	1.919292704	0.007662391
Ubap1l	1.918047658	2.55E-06
Slc30a2	1.915355913	0.002213606
Ret	1.911849255	3.00E-14
Adra1d	1.908875948	7.95E-26
Trim21	1.908519864	2.67E-74
RT1-CE1	1.901489409	1.51E-39
Arpp21	1.890441238	2.45E-16
Hecw1	1.88958464	5.02E-12
Kif5a	1.883342797	3.67E-22
NEWGENE_1565644	1.881148083	0.028545616
AABR07059663.1	1.875205219	9.76E-69
AABR07044414.1	1.873108638	1.67E-15
Kcnq3	1.870680608	3.95E-05
Dyrk3	1.86741422	1.64E-23
Tmprss5	1.862960403	0.045769415
LOC100912489	1.861418096	9.90E-06
Nefm	1.859406496	2.93E-11
Rhov	1.843393487	0.005191298
AABR07004945.1	1.841335073	0.045195581
Spata31d1	1.817781004	0.00931224
RT1-T24-4	1.813789129	1.03E-59
RT1-CE7	1.808423385	2.48E-40

Cnksr2	1.802431128	2.92E-11
Eif2ak2	1.797789661	6.41E-108
Ptchd1	1.796289225	6.58E-24
Ifi27l2b	1.786647468	8.28E-24
Ampd1	1.786128606	0.002548285
Krtap3-2	1.784501525	0.038064748
Etfbkmt	1.781324308	1.67E-82
AABR07024139.1	1.774550844	0.044373662
Parp10	1.772123125	5.19E-49
Stat2	1.771237438	3.03E-108
Tmem163	1.771181735	8.35E-09
Rnf152	1.770798611	1.13E-23
Zfp385d	1.769112456	4.45E-34
Ros1	1.766432547	8.97E-12
Mitd1	1.76063452	1.01E-35
Sema3a	1.758445496	4.62E-13
Ppp1r1b	1.757935148	0.002572914
AABR07054319.1	1.757351274	1.09E-31
Plppr3	1.755804861	0.002292497
Vax2	1.755566903	0.000120614
Irf9	1.752354983	1.07E-22
Syt9	1.741839879	4.59E-06
Slc24a3	1.740685667	8.35E-43
AABR07042077.1	1.734480664	0.029924104
Hpx	1.728377712	4.01E-14
Slc47a1	1.721320064	0.007956722
Gbp2	1.716791918	7.99E-43
LOC298139	1.715868544	8.90E-19
Ccne2	1.71519005	2.70E-117
Nos2	1.71258693	2.61E-38
Dmrt2	1.709799279	0.015631707
Clstn2	1.704608769	3.30E-22
AC128859.3	1.703844545	6.34E-05
RT1-CE10	1.702636915	3.83E-40
AABR07058464.1	1.699560602	3.76E-30
Ifi35	1.698942589	1.32E-37
Psmb8	1.697536415	4.05E-24
Nox1	1.69028051	1.34E-05
Ccl4	1.687267891	1.37E-13
LOC100911562	1.681731565	2.82E-23
Npas4	1.681022546	1.70E-06
LOC100359515	1.67330055	4.80E-21
Cacng4	1.673015304	3.14E-07
AABR07068214.1	1.671623173	2.53E-16
RT1-A2	1.669861553	4.83E-38
Six1	1.656828689	2.65E-12
AC119015.4	1.646446734	4.74E-12
LOC691215	1.644709159	8.60E-07
Duox2	1.63055521	8.65E-06
Usp51	1.630108252	1.64E-06
RT1-T24-1	1.625662665	9.95E-25
Zc3hav1	1.624643725	1.17E-96
Tapbp	1.623270474	9.19E-67
Pou3f1	1.622726936	0.013170657
Efnb2	1.620460035	1.70E-165
Neto2	1.619919024	1.10E-38
Mmp12	1.618469528	1.49E-17
AC128962.1	1.615566283	5.58E-09

AABR07054554.1	1.613418798	0.000210203
Slc35f2	1.610505891	9.23E-18
Dnajc6	1.604535181	0.000773426
Tex15	1.602628259	1.23E-07
RGD1309808	1.602333348	3.86E-24
Mybl2	1.602089234	4.92E-122
Diras2	1.601841989	0.001040316
Tent5c	1.600868417	1.17E-11
Fam83g	1.600788614	0.024100883
Slitrk5	1.600155961	1.15E-18
Socs1	1.599116247	2.76E-25
Cxcl11	1.598537932	2.73E-05
Pcsk1	1.591395061	0.049137522
AABR07060833.1	1.589455093	6.17E-09
Bdnf	1.58883653	1.54E-41
Stat1	1.584891134	7.43E-121
Sp100	1.582526762	4.67E-33
Plppr4	1.580811045	5.91E-25
Trim25	1.577940088	2.80E-68
Prrt4	1.577568722	3.03E-05
Calcrl	1.575374773	2.39E-55
Cfb	1.573944861	1.37E-21
Ccdc36	1.573326985	4.00E-06
LOC102551929	1.567626661	0.014867784
Gabra4	1.565582632	3.21E-14
Trim34	1.565038934	4.64E-43
AC127084.5	1.5614517	6.17E-11
Ifi27	1.559670817	7.83E-89
Armh4	1.556522491	5.22E-13
AABR07044383.1	1.551037311	2.95E-18
Bcl11b	1.549197665	1.61E-17
Gnrh1	1.546138954	0.008973944
Car2	1.540625512	1.21E-39
Lancl3	1.535245003	0.004726054
Sall1	1.533575043	6.34E-05
Tent5a	1.53332526	3.70E-105
Nefh	1.532935954	1.41E-38
RT1-CE4	1.531611275	2.90E-32
RT1-CE3	1.530335737	2.16E-17
Gpr21	1.52832707	0.026637421
LOC100364500	1.525741929	7.67E-15
Sp110	1.520845531	3.17E-49
Clstn3	1.518506111	0.015606054
Cgas	1.518299402	1.03E-15
RT1-S3	1.517860949	5.15E-50
Cds1	1.51723407	0.000145413
Ly6e	1.516275787	8.76E-113
Kcnk1	1.515032153	5.13E-05
LOC685067	1.510942295	5.12E-66
Stk32a	1.508345072	0.001430722
Tas1r1	1.499641467	1.62E-10
Mefv	1.497591355	0.013118052
AABR07044404.1	1.497084701	4.40E-13
Kcnj3	1.493863552	2.32E-21
Slc52a3	1.492812121	0.026008162
RT1-A1	1.491986326	2.51E-28
Sytl2	1.491317968	6.46E-13
Cd109	1.490817552	2.78E-52

Cdk5r1	1.487832366	0.003672293
Sord	1.48682227	8.53E-85
AABR07044421.1	1.480469401	2.18E-14
Fen1	1.480308415	1.42E-86
Wscd2	1.48029321	7.62E-24
Slc29a4	1.476150047	1.05E-11
Bicdl1	1.471614543	1.02E-12
LOC108348215	1.467509103	0.000984562
Ube2l6	1.465320706	1.04E-31
Gch1	1.463459175	8.80E-10
Cd55	1.461093183	7.58E-87
Lgi2	1.458360514	6.98E-08
Gata3	1.45531408	1.16E-07
Slc35g1	1.453589735	7.13E-05
Map2	1.45178066	1.95E-17
Gjb3	1.438988096	5.05E-11
Enkur	1.43698046	3.39E-05
Ch25h	1.43693559	1.38E-07
Slc16a10	1.435523619	1.10E-11
Foxf1	1.434767504	1.30E-08
AABR07044468.1	1.433911376	0.039543904
B3gnt5	1.430687162	1.86E-16
Adra2a	1.429930708	2.77E-17
Sctr	1.422745755	0.020125229
Gabrb3	1.422205146	0.015188325
Khdrbs2	1.420087446	1.82E-09
Brinp2	1.413846016	5.03E-07
Irf4	1.412405737	0.042402958
Apcdd1	1.411978343	2.57E-47
Cntn4	1.4047676	0.042159081
AABR07035839.1	1.402513264	6.01E-06
Sema3e	1.40214377	2.81E-06
Fam181b	1.400863287	0.000106903
Cdh18	1.40021115	0.000313117
Zfp385b	1.383806858	2.44E-10
Htr1b	1.378054393	0.001122969
Mmp16	1.373929556	1.63E-29
AABR07049695.3	1.371911429	3.04E-05
LOC100912195	1.371761249	1.10E-59
Znfx1	1.371706974	9.10E-48
Nkx6-1	1.368604661	0.001988049
Mov10	1.367694626	8.25E-56
Resf1	1.367165926	1.50E-117
LOC500584	1.366764707	9.56E-60
7SK	1.366095571	0.005259888
AC118772.2	1.363557884	2.92E-05
Hcn1	1.363017761	0.023773491
AABR07006367.1	1.36015573	0.025884755
Cdh24	1.358774987	7.10E-35
Ppm1n	1.358548639	0.00288166
Snta1	1.357421366	8.45E-107
Pml	1.35596793	6.39E-90
Vamp1	1.352411405	3.18E-61
Hspa1b	1.35172783	6.05E-60
Nabp1	1.351427823	5.74E-43
Actr3b	1.350470246	7.95E-21
Syn1	1.348859327	2.12E-12
Tes	1.345821195	1.21E-20

AABR07024203.1	1.345625508	2.68E-05
Slfn2	1.334852462	2.22E-21
Caskin1	1.333703081	1.13E-24
LOC108348108	1.330901511	3.66E-53
Spata18	1.323523199	4.06E-05
Lonrf3	1.322709493	1.22E-20
Cdkn1c	1.321410524	1.52E-11
Gbp6	1.318761103	5.38E-14
Tmem106a	1.318163492	6.80E-16
Amigo2	1.317291287	1.04E-27
Has3	1.31500576	5.60E-13
AABR07039334.1	1.313404411	5.29E-08
AABR07035916.1	1.310193059	0.026983038
Ccl7	1.302307623	1.02E-21
AABR07058124.4	1.298480292	0.000127008
Cdca7	1.29575118	8.62E-34
RT1-CE5	1.29550851	1.78E-56
Zfp711	1.29231003	6.13E-13
Csf3	1.291612834	3.04E-05
AC120486.3	1.289402996	0.000263019
B3gat1	1.28816876	3.45E-10
RT1-N3	1.287148891	7.29E-33
Fam20a	1.286075774	4.02E-19
Tonsl	1.285417884	5.14E-32
AABR07063126.1	1.281313879	0.018348544
Sytl2	1.280211036	1.18E-21
Slc16a4	1.274475161	0.000274157
Sema7a	1.272616204	2.76E-22
Ca8	1.271739706	6.90E-21
Efna2	1.271366625	1.21E-05
AABR07068221.1	1.268974936	3.74E-06
Lef1	1.268213895	9.76E-34
Npnt	1.266069322	4.49E-23
AABR07035796.1	1.261434844	2.28E-08
AABR07044362.4	1.260042481	2.53E-15
Il15ra	1.258728381	4.15E-06
Ccdc85a	1.258622236	7.38E-08
Cldn23	1.258464424	0.003026728
Plcb1	1.258327546	8.37E-16
Cxcl6	1.25719596	8.04E-08
Prdm8	1.257052324	1.72E-06
Slfn13	1.255564416	5.60E-108
Trim24	1.255020384	5.99E-47
LOC498675	1.253262152	5.79E-05
Sncg	1.251070258	3.13E-17
Il17re	1.248904831	5.05E-08
AC120486.9	1.247120585	2.49E-07
Pbld1	1.246598726	0.00078154
Pparg	1.244839109	8.09E-07
Prkg2	1.23773485	0.009738634
Rasa4	1.237554231	2.45E-12
Asb16	1.235822216	0.004308287
Tmtc1	1.23576443	1.44E-33
RGD1560010	1.235635019	9.85E-31
7SK	1.234480099	0.029561242
Plcg2	1.234040853	1.80E-30
Shfl	1.231756926	5.77E-26
Sema6b	1.226526981	0.021258007

Prima1	1.223407366	9.45E-18
Ptchd3	1.215319314	0.000103313
Ccl3	1.212648522	1.46E-15
Pou2f3	1.211905464	2.25E-05
Il2ra	1.210041984	0.039568235
Cdk2	1.208025716	8.20E-98
Kcna3	1.202701691	0.00089483
Tgfa	1.198351279	3.93E-34
Kcnk5	1.198159146	1.06E-05
AABR07045485.1	1.196319177	5.00E-13
Nsg1	1.196107663	6.55E-08
Rtl9	1.195232231	0.016016108
Ccr1	1.195097663	0.027185898
Xk	1.195068097	1.11E-12
Tsga10	1.192478866	2.38E-08
Dnd1	1.191981783	0.001678649
Atp2b2	1.187315879	5.02E-12
Tmem151a	1.185914099	5.55E-07
Ccne1	1.185469181	2.61E-41
Asb15	1.182742065	9.20E-10
Rbm43	1.182205256	4.98E-12
Tlr7	1.181537795	4.26E-05
Map3k9	1.179461422	0.000132936
Fras1	1.179306018	2.62E-06
Lrr1	1.179013925	0.002445843
Alox5	1.173857443	1.60E-08
Hcn2	1.173397168	5.49E-36
Itga6	1.172155006	3.81E-76
Mcm3	1.171494405	1.44E-100
Tmem196	1.170345947	2.07E-20
Pcdh9	1.165660957	6.04E-09
Gpat3	1.16407819	8.63E-44
Pcdh17	1.163766756	1.58E-16
Hap1	1.162888086	4.39E-26
Eps8l2	1.162632363	0.002107851
Lgals5	1.160651625	5.46E-13
Kcnc1	1.160421902	0.000208659
Slc6a15	1.157016467	4.58E-07
LOC292543	1.155566499	2.62E-37
Klf14	1.153465115	0.015454298
Nfkbiz	1.153257042	0.001039815
Cttnbp2	1.1526005	5.59E-07
Il1a	1.151657669	1.99E-05
Tchh	1.151306146	3.97E-08
Corin	1.147488068	0.006423018
Hdac9	1.146726725	9.38E-31
Dlgap3	1.146541793	0.00016429
RT1-M6-1	1.142475364	5.55E-06
Wdhd1	1.139499374	1.54E-56
Mcm6	1.138870292	6.67E-132
Arl4a	1.138071823	7.17E-31
Rubcnl	1.136669093	3.13E-06
Hs3st1	1.134693619	5.68E-15
C4a	1.131823908	8.13E-19
Dnajc9	1.13041159	1.70E-54
Trim36	1.129073168	8.98E-07
Iqgap2	1.128754454	3.39E-12
Pinlyp	1.128546858	1.09E-07

Maoa	1.127271883	1.53E-22
AABR07028979.1	1.126473522	4.11E-10
Hmgb2	1.124774504	2.14E-42
Ntng1	1.123819988	4.97E-13
Nmi	1.122692962	8.03E-11
Lgals9	1.122558097	1.81E-10
Dgat2	1.117174781	1.36E-42
Sh3bgrl2	1.111051432	7.26E-05
Ntf3	1.10994997	5.50E-14
Aff2	1.109679057	4.45E-14
Sox11	1.108814767	3.54E-17
Abhd3	1.108458212	7.63E-05
LOC103689965	1.108215412	1.18E-14
E2f8	1.107089986	1.02E-28
Elavl4	1.105130815	0.000153625
AABR07017145.2	1.104544479	2.99E-13
AABR07015743.1	1.103490502	1.63E-12
Akr1c15	1.102395654	3.14E-15
Kcnq5	1.102290769	6.42E-30
Cadps	1.102124751	1.71E-20
Bpifb5	1.09869323	8.64E-13
RGD1559962	1.096843862	1.98E-15
Adcy3	1.094691371	1.57E-27
Gprin3	1.094681438	0.000377415
Plcl2	1.093740388	3.99E-46
Mctp1	1.093207506	2.60E-09
Tshr	1.093089667	0.001127309
Dclk2	1.082367711	1.57E-55
AC120486.8	1.082217885	1.61E-11
AABR07026112.2	1.079346531	0.029419657
Lrrc7	1.078587782	0.000441215
Tap2	1.076932408	3.27E-13
Wnt6	1.075648647	0.023278331
Hmgb2l1	1.07518459	3.90E-20
Sfmbt2	1.073372696	6.85E-05
Ldlrad3	1.072921079	1.84E-46
Kcnj12	1.071296063	2.58E-15
Abhd15	1.071162734	0.004238314
Chrna5	1.066238417	2.61E-09
Slc9a5	1.065664028	7.43E-06
Lratd2	1.064532893	6.67E-05
Asf1a	1.0616458	2.36E-73
Hells	1.061155591	5.63E-11
Ccdc96	1.060358545	0.01733265
Six2	1.058391808	0.000102432
Epha3	1.057077861	2.08E-25
Thbd	1.057015926	8.05E-18
LOC103689996	1.05680256	7.22E-13
Sap30	1.056424217	5.18E-21
Frmd3	1.056029793	0.000151264
Arrb2	1.055465891	7.02E-20
LOC102549471	1.055340295	6.49E-05
LOC100911660	1.052059342	1.45E-15
Mtss2	1.051077317	9.30E-31
Stac2	1.050441733	3.96E-11
Cbx4	1.049991508	3.14E-48
Fam89a	1.049962338	3.39E-06
Itgb8	1.049082034	7.41E-12

Cbln2	1.04521048	1.98E-06
Usp43	1.043670884	3.33E-07
Zbtb16	1.04356418	0.000211824
Tnf	1.042888312	1.70E-05
Ddias	1.04050024	5.07E-22
Them6	1.040169509	5.83E-31
Neurl3	1.040158532	0.005993675
Itpka	1.036578754	0.000586973
Nudt10	1.03586827	7.11E-10
AABR07047256.1	1.034526905	1.21E-06
Rell1	1.032257833	0.000244066
Cxcl2	1.031898198	1.94E-06
Crym	1.03067011	6.14E-44
Cd274	1.02815882	6.26E-05
Zfand2a	1.02751382	1.25E-80
AABR07053741.1	1.025803053	2.27E-32
Pde5a	1.024375731	2.69E-10
Nanos1	1.02306425	1.80E-08
Khdrbs3	1.021729364	5.88E-30
Plin4	1.02090403	2.54E-12
Asb4	1.020138765	1.69E-39
Abcd1	1.020106124	2.23E-53
Cpeb2	1.019855618	1.04E-24
Rnf227	1.017522431	7.07E-05
Pgm2l1	1.016374106	1.92E-16
LOC103690108	1.014820072	1.23E-17
Kcnc4	1.014621149	0.036987993
Fam83h	1.012772574	8.47E-11
Gltpd2	1.012057227	0.040431879
Cmtm8	1.011664685	0.000920291
Slc1a1	1.011041837	5.06E-07
Ascc3	1.010174845	1.64E-29
Ccdc68	1.009380128	0.000121832
Tnfsf9	1.007170271	0.005069039
Mlkl	1.006167258	1.00E-36
Rpa1	1.006038019	4.28E-94
Fbxw17	1.005189471	2.16E-23
Gldc	1.003404336	0.001085481
Rab26	1.003114377	5.29E-08
Tnfrsf19	1.002946581	3.43E-08
Gpd1	1.002194317	9.51E-19
B2m	1.001242225	2.33E-22
Lck	1.000657508	0.004671723
Trappc6a	-1.000099967	6.96E-12
Spink8	-1.003298426	4.36E-11
Tlcd2	-1.005325491	4.10E-05
Gata4	-1.007883587	5.89E-73
Hddc3	-1.01139204	4.04E-35
Hoga1	-1.012460962	0.007301685
Mlip	-1.012526778	5.89E-45
Cdc42bpg	-1.015809597	1.83E-32
Ryr2	-1.015920146	3.75E-09
RGD1561662	-1.016949389	0.013619982
Olfml1	-1.017943367	1.92E-09
Efnb3	-1.019642366	1.24E-30
Amn1	-1.019764233	2.34E-17
Dmac2l	-1.020852665	5.39E-09
Cdh3	-1.022048416	2.01E-30

Slc1a7	-1.023309753	0.00544032
Dync2li1	-1.024636837	3.92E-08
Slc45a3	-1.025308733	0.009889275
AY172581.18	-1.025855222	7.99E-66
Gstm7	-1.027232832	3.87E-09
Fdft1	-1.027972235	1.63E-100
Azin2	-1.028605238	1.56E-13
Myl4	-1.029710644	4.22E-95
Tlr5	-1.030662484	1.70E-06
LOC100294508	-1.033668645	1.41E-89
AY172581.10	-1.035305739	3.91E-49
Myl3	-1.035331675	2.42E-25
Wdr27	-1.036829702	0.046536241
Micall1	-1.037995911	1.99E-77
Lamc2	-1.040095731	1.72E-13
Nprl3	-1.040417717	1.08E-25
Crb3	-1.04071415	0.005994079
Srpk3	-1.041792146	5.80E-34
Itga9	-1.042887269	1.59E-28
Prkcb	-1.046588734	0.000473416
Prelid2	-1.049116538	1.74E-11
Iglon5	-1.051181335	1.30E-10
Kctd13	-1.051521977	2.23E-12
Etfb	-1.052963561	2.09E-50
Alpk2	-1.053898502	1.95E-42
Atp1a2	-1.055690114	1.55E-13
Aqp1	-1.056458897	6.76E-150
AABR07020987.1	-1.056461375	0.016408098
Phyh	-1.057224214	5.63E-85
Morn4	-1.060705648	2.81E-13
Fxyd1	-1.062944472	3.80E-24
Selplg	-1.063661917	2.25E-27
Lurap1	-1.067007813	0.001061227
Sh3bgr	-1.06842334	1.13E-26
Arrdc4	-1.068638827	3.05E-21
Vstm2b	-1.069080855	0.041104784
Hsf2bp	-1.069518162	0.000109457
LOC100911951	-1.069541481	0.033757094
Ntpcr	-1.071369752	4.44E-13
LOC103689954	-1.073176065	0.00014435
Clip4	-1.074870811	1.40E-35
AABR07021022.1	-1.076818641	0.005973595
LOC102553613	-1.080260101	2.13E-26
Clec4a1	-1.080575484	0.001269199
Abi3bp	-1.081210366	0.001323543
Mylk3	-1.08133679	6.46E-59
AABR07058441.1	-1.085154968	0.004354885
Tmem150a	-1.088034695	1.99E-70
Scn7a	-1.089380738	0.000272092
Hspb7	-1.091033085	3.05E-62
Gpr75	-1.092687376	2.23E-05
Mipep	-1.100210633	9.57E-44
Unc119	-1.100216853	2.99E-53
Cpa6	-1.10142671	0.011273703
Brsk1	-1.101504363	7.53E-41
Pm20d1	-1.108661696	6.10E-18
Gipr	-1.109206251	8.58E-05
Tac3	-1.11358122	1.13E-05

Klhdc8b	-1.113862043	2.68E-70
Kcnip2	-1.11465114	0.02771088
Lrrc4b	-1.114973976	2.34E-11
Fgf18	-1.115495002	8.73E-27
Socs2	-1.11672547	2.95E-37
Spon1	-1.117347616	1.74E-44
Tst	-1.11792582	1.36E-42
AC105662.2	-1.124329784	0.016401196
Ccl20	-1.12618743	9.80E-24
Adhfe1	-1.126956974	5.89E-05
Fggy	-1.1311807	1.19E-05
AY172581.7	-1.13515951	5.36E-39
Slc25a26	-1.135622883	7.59E-53
Ift43	-1.13587614	1.26E-58
Pard6b	-1.135921732	1.15E-16
Mapt	-1.138122648	0.012215576
Adssl1	-1.140802604	1.68E-17
Efemp1	-1.14288914	3.41E-05
Sorbs2	-1.148343873	5.42E-69
Tnni3	-1.14938409	3.81E-70
LOC100911486	-1.149538865	8.08E-12
Adamtsl3	-1.150460507	1.93E-06
Nt5m	-1.151861291	1.18E-07
Sardh	-1.154625074	9.52E-31
Fam189a2	-1.155223168	5.13E-56
Cox6a2	-1.159067539	3.54E-29
Lrrc17	-1.159876851	2.58E-45
Ppl	-1.166528463	3.05E-06
Ntrk2	-1.168610493	3.27E-28
Cyp1a1	-1.169293	0.005102287
Ntsr2	-1.17173692	0.049064588
Scn5a	-1.172227172	6.85E-52
Scfd2	-1.17836108	2.94E-32
Tbx20	-1.180246379	1.67E-72
Lrrn4	-1.181580263	0.000457165
Tmem100	-1.18541544	1.76E-05
Mir27b	-1.188586574	0.042930301
Ldlr	-1.189413344	1.63E-42
AABR07030086.2	-1.190817592	0.007232033
Tmem176a	-1.190860639	2.14E-11
Synpo2l	-1.191419231	3.34E-92
Frem1	-1.191823242	2.27E-09
Lyrm9	-1.191901141	6.55E-14
Flt4	-1.193113168	2.79E-11
Smtnl2	-1.195485754	2.34E-14
Trim55	-1.197210353	5.31E-127
Fbxo16	-1.197975662	0.031427768
Dtna	-1.199705082	1.39E-67
Svip	-1.200126347	2.48E-08
Col8a2	-1.20649001	8.57E-14
Actn2	-1.210121775	2.11E-97
Atp1b2	-1.21297319	9.54E-28
Samd12	-1.213138511	5.33E-06
Epb41l4b	-1.213747593	1.16E-45
Wnt5a	-1.214019823	6.13E-54
Mfap2	-1.218585986	2.78E-19
Nckap5	-1.220742005	1.50E-10
S1pr1	-1.223859717	4.15E-75

Akap7	-1.227507813	1.19E-10
Stc1	-1.228176864	1.07E-08
Sash3	-1.229182174	0.017724162
Hhatl	-1.23468319	3.02E-75
AABR07037243.1	-1.243311205	1.20E-105
Syngr1	-1.244956593	8.83E-09
Sycp3	-1.245858148	0.00078288
Crip2	-1.246799584	1.27E-122
Hsd17b7	-1.249323789	2.78E-51
Akr1c14	-1.250600091	0.000256021
AABR07070310.1	-1.253231489	1.08E-12
Fbxl22	-1.254874523	2.05E-50
Nwd2	-1.255861903	0.004968762
Tmem106c	-1.259574847	5.94E-28
Sbsn	-1.27205581	3.69E-09
Bphl	-1.277494777	1.95E-15
Dnaaf3	-1.280517745	0.011625557
Tbc1d10c	-1.282746364	2.74E-06
Scn10a	-1.294655284	2.55E-12
Krt19	-1.295439116	2.91E-06
Cytl1	-1.30338212	0.010998747
AABR07052585.2	-1.308866916	7.32E-15
Myl7	-1.317189637	1.32E-43
Ndufaf7	-1.320133945	5.95E-31
Sspn	-1.323474208	6.70E-15
Fam163b	-1.328355077	0.000479355
Tmod1	-1.328690697	2.20E-148
Myom1	-1.344651499	9.38E-88
Tmem150b	-1.356083657	0.019925949
Clec4a3	-1.366184131	0.043474968
Scd	-1.369174543	2.45E-57
Trim72	-1.371751143	1.19E-17
Cd74	-1.38171994	0.019136095
Fgf16	-1.396521875	1.37E-29
Tnni3k	-1.40292142	3.84E-74
Lbh	-1.402982554	1.48E-107
Egr1	-1.408054835	8.55E-73
Fcgbp	-1.412061738	3.29E-16
Myh7b	-1.420995619	5.40E-12
Hmgcs2	-1.430354034	1.87E-07
Arhgef39	-1.433162492	2.64E-107
Fdps	-1.44094644	3.31E-69
Dpf3	-1.443879637	5.52E-19
Dpp4	-1.454261925	1.19E-08
Efcab6	-1.455516959	6.66E-37
AABR07012058.2	-1.465466266	3.67E-54
Tril	-1.468180747	1.20E-72
Col6a6	-1.470674084	1.33E-12
Cacna1s	-1.47643834	2.59E-14
Pla2g5	-1.478030836	3.47E-123
Tnni1	-1.48622245	1.02E-116
Itgb1bp2	-1.486453531	3.05E-63
AABR07064753.1	-1.489580961	4.62E-08
Mybpc3	-1.490695452	1.14E-95
Tnnc1	-1.496293335	1.67E-47
Actc1	-1.496508136	6.01E-35
Msln	-1.497133118	3.88E-10
LOC681355	-1.503853723	0.007830141

Gnb3	-1.506398368	5.01E-13
Hamp	-1.510321303	0.000157098
Ldb3	-1.512895786	9.83E-120
Ccdc107	-1.516569002	4.89E-136
U1	-1.518051766	0.04201078
Slc13a3	-1.519509194	0.00021046
Igfbp2	-1.520795429	6.64E-10
AABR07030443.2	-1.544461837	2.50E-22
Myh6	-1.544562158	9.33E-99
Tmem179	-1.553349074	1.72E-09
S100a1	-1.559584963	2.32E-22
Epn3	-1.567652004	5.05E-48
B4galnt4	-1.570035543	2.55E-12
Fignl1	-1.574499491	9.91E-179
Fn3k	-1.577190329	0.00397885
Phf24	-1.590690686	4.29E-45
Dlk1	-1.591817917	3.57E-41
Adamtsl2	-1.593926425	5.30E-37
Inha	-1.616735955	6.72E-14
Fstl3	-1.62956084	5.79E-63
Mybpc2	-1.640830724	4.88E-33
Dpysl4	-1.641039002	0.029809313
Rab3a	-1.657580842	1.62E-54
Serinc2	-1.66167157	5.58E-86
Kcnq1	-1.698481251	5.42E-72
AABR07054614.1	-1.724653486	2.13E-66
Fads6	-1.726017187	2.82E-15
Tmcc2	-1.731187115	5.83E-43
AABR07034131.1	-1.73762337	1.06E-15
Mb	-1.756632142	1.94E-29
Csrp3	-1.77862358	5.26E-101
Klhdc8a	-1.796622533	0.001888703
Myh3	-1.810657714	4.81E-30
Syt12	-1.812857095	4.05E-18
Colca2	-1.828859267	0.047503263
G0s2	-1.83760994	1.00E-69
Irx4	-1.845076436	1.48E-54
Pirt	-1.846930215	0.000658646
Cavin4	-1.846937176	8.52E-65
Ppp1r1a	-1.84812523	1.57E-32
AABR07025743.1	-1.851432855	9.98E-05
Cacna1g	-1.856355107	3.15E-131
Smim5	-1.865828669	2.78E-09
Tmem71	-1.866128576	4.93E-28
Kcnn1	-1.868804879	6.44E-13
Tle6	-1.883740656	0.000103426
Gramd2a	-1.885975234	1.43E-08
Col26a1	-1.898638533	0.001027405
Inka1	-1.924548121	0.000235198
Mir675	-1.933383966	2.98E-11
LOC103690354	-1.949728659	0.041929113
Lamb3	-1.952909423	1.49E-286
Acot11	-1.977216296	1.07E-05
Syne4	-2.06548501	0.031717577
AC111885.1	-2.076543545	0.045125129
Myh7	-2.122631947	1.38E-142
Nppb	-2.141067194	2.35E-27
Nppa	-2.148655062	4.48E-35

Calhm5	-2.161924802	0.027067041
U6	-2.182980432	0.005231603
Myom2	-2.194693519	2.32E-31
Kl	-2.198125647	0.00329274
Nsg2	-2.226549861	8.45E-49
Sult5a1	-2.233978777	0.003592282
Septin1	-2.240686739	0.015238225
AC135026.1	-2.280724216	0.004979145
Epb42	-2.295252218	0.018321025
Ankrd1	-2.308791866	3.01E-177
Sfrp5	-2.325329188	0.027911162
Myl2	-2.3480828	3.79E-110
Fcrl2	-2.411852216	6.05E-17
Ckmt1	-2.422993612	1.78E-45
Aldoc	-2.433199193	4.75E-27
Lrtm2	-2.510349801	0.002707434
Krt17	-2.54949793	0.000336173
Papln	-2.613171316	0.021654076
Lcn12	-2.637102382	0.031704089
Mrap	-2.744852827	0.040911752
RGD1308544	-2.757355889	0.007145997
Slc26a10	-2.772965609	0.014015653
Tnnt3	-2.840290094	0.04366278
Aspdh	-2.846171502	4.94E-06
Gp1bb	-2.963544012	6.01E-05
Cpa2	-2.986641871	0.003135581
Dmp1	-2.999196138	0.022280822
Trpm8	-3.115308468	0.000815922
AABR07018064.1	-3.24975987	0.000240371
Pld4	-3.300327138	0.004431108
AABR07058410.1	-3.427129617	0.043359433
C1qa	-3.508806024	0.00468663
Ackr2	-3.598546806	0.022209446
Spata20	-3.772508776	0.000283156
Myo5c	-3.915224066	4.12E-06
Rorc	-4.326958457	0.041104784
Fgfbp1	-4.540419307	0.024030106
AC114452.1	-4.611973877	0.009483737
AABR07036452.1	-4.674208059	0.035218061
AABR07006269.1	-4.746375336	0.028956266
LOC360479	-4.75405185	0.028485905
Blk	-4.762242072	0.027980033
Aqp4	-4.763655549	0.033471114
Tnni2	-4.774550007	1.34E-10
Serpina11	-4.810789889	0.024205437
Cmtm5	-4.823088572	0.023671924
Adad1	-4.929408512	0.016195963
Myoz3	-4.943119647	0.01512107
Ryr1	-5.15064309	0.042127277
Fabp1	-5.151748389	0.041507459
Krt24	-5.238433691	0.0052183
AABR07014974.1	-5.247197101	0.000274866
LOC689986	-5.387438254	0.042336281
Clvs1	-5.574099701	4.51E-05
Tnnc2	-5.908413542	8.78E-06
Col23a1	-5.967179954	1.95E-06
Fam71e1	-6.540456108	2.52E-08
Cpa1	-6.732300066	1.00E-08

Ermap	-6.751296972	5.30E-09
Pkd1l2	-6.93186008	0.010035146

Supplemental Table 8. Sequence information of qPCR primers used in this study

Species	Gene name	Forward primer (5'→3')	Reverse primer (3'→5')
mouse	Mettl5	AACTAGAGAGTCGCCTGCAAG	CTGCAACCGCTTTGTTTTCAA
mouse	Nppa	CACAGATCTGATGGATTTCAAGA	CCTCATCTTCTACCGGCATC
mouse	Nppb	GTCAGTCGTTTGGGCTGTAAC	AGACCCAGGCAGAGTCAGAA
mouse	Acta1	GCCCATCTATGAGGGCTATG	AATCTCACGTTCAGCTGTGG
mouse	Col3a1	CTGTAACATGGAAACTGGGGAAA	CCATAGCTGAACTGAAAACCACC
mouse	Col1a1	AATGGCACGGCTGTGTGCGA	AACGGGTCCCCTTGGGCCTT
mouse	Fn1	GGAATGGACCTGCAAACCTA	GTAGGGCTTTTCCCAGGTCT
mouse	Postn	TGTGTATCGGACGGCTATCT	CTCTGCTGGTTGGATGATTTCT
mouse	β-actin	GTGACGTTGACATCCGTAAAGA	GCCGGACTCATCGTACTCC
mouse	GAPDH	AGGTCGGTGTGAACGGATTTG	TGTAGACCATGTAGTTGAGGTCA
mouse	18S	TCCGACCATAAACGATGCCG	CAATCTGTCAATCCTGTCCGTGTC
rat	Mettl5	GCAAGAAGTGGATGGATTCG	TCGTCGTATGTATTGTGGATTGT
rat	Nppa	CAACACAGATCTGATGGATTTCA	CCTCATCTTCTACCGGCATC
rat	Nppb	GTCAGTCGCTTGGGCTGT	CCAGAGCTGGGGAAAGAAG
rat	Acta1	AGCTATGAGCTGCCTGACG	GATCCCCGCAGACTCCATA
rat	Suz12	TCGACAACCTGGATTTGCCTT	CCAGAAACTCCGACATGCTT
rat	Mef2a	GCACTACAGACCTCACGGTA	CCTACACTATTCGCACCAGT
rat	Mef2d	AGTACGCCAGCACCGACA	TCACAGCCGTTGAAACCCTT
rat	β-actin	CCCGCGAGTACAACCTTCT	CGTCATCCATGGCGAACT
rat	GAPDH	GATGCTGGTGCTGAGTATGTCG	GTGGTGCAGGATGCATTGCT
human	METTL5	AAGGAACTAGAGAGTCGCCTG	GCGGCCTGGTAGGATACTG
human	GAPDH	AGATCCCTCCAAAATCAAGTGG	GGCAGAGATGATGACCCTTTT

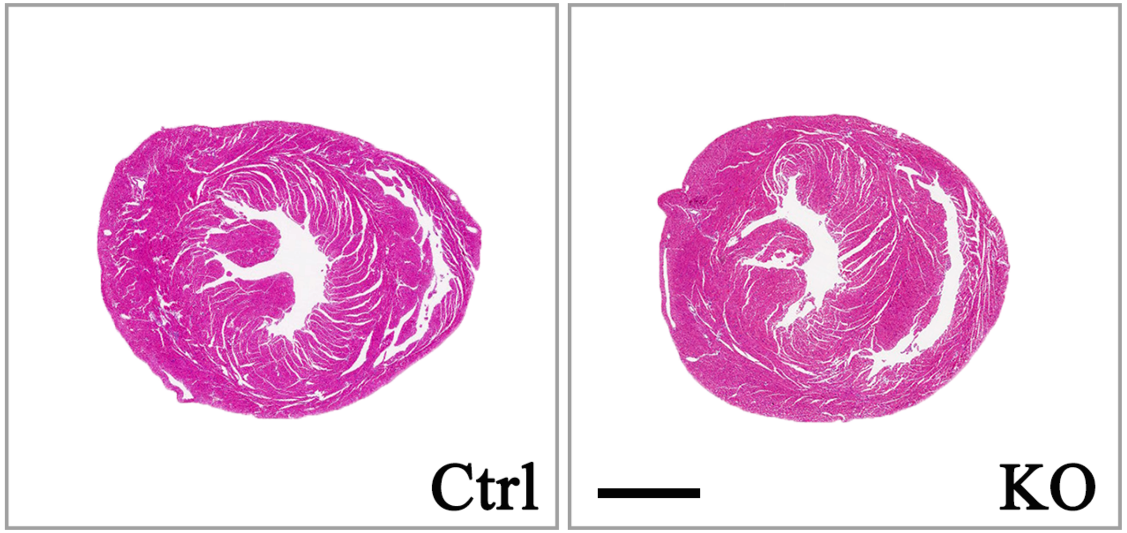

Supplemental Figure 1. Representative images of H&E staining of 1-month-old Mettl5-KO and control hearts. Scale bar=1 mm.

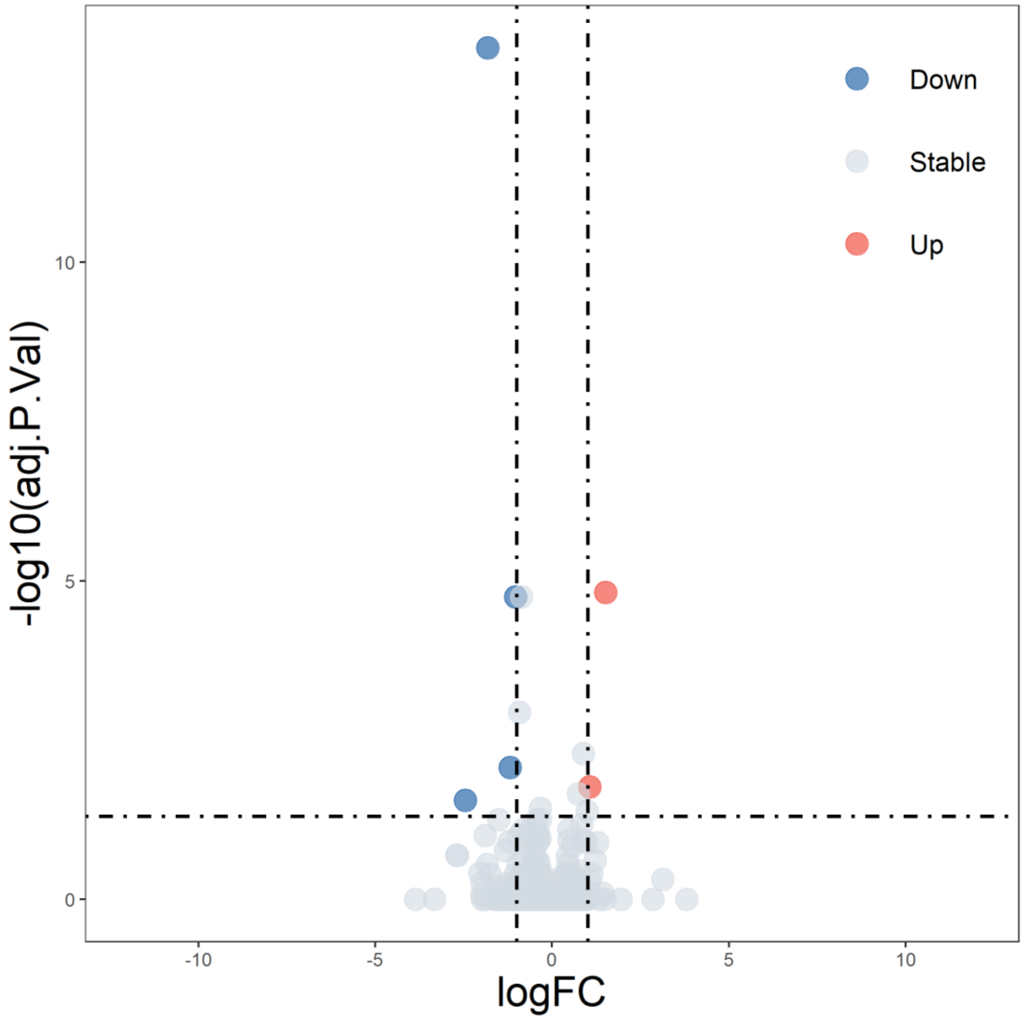

Supplemental Figure 2. Volcano plot of differentially expressed genes in 1-month-old Mettl5-KO and control hearts. Only 6 dysregulated genes are found between groups (|Log2FoldChange|>1, adjusted P Value <0.05).

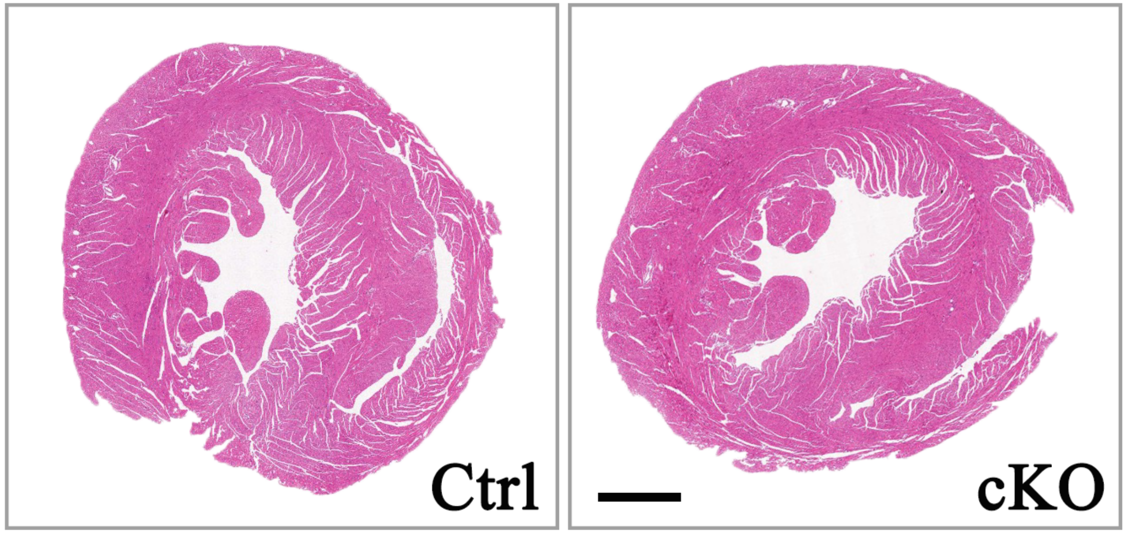

Supplemental Figure 3. Representative images of H&E staining of 12-week-old Mettl5-cKO and control hearts. Scale bar=1 mm.

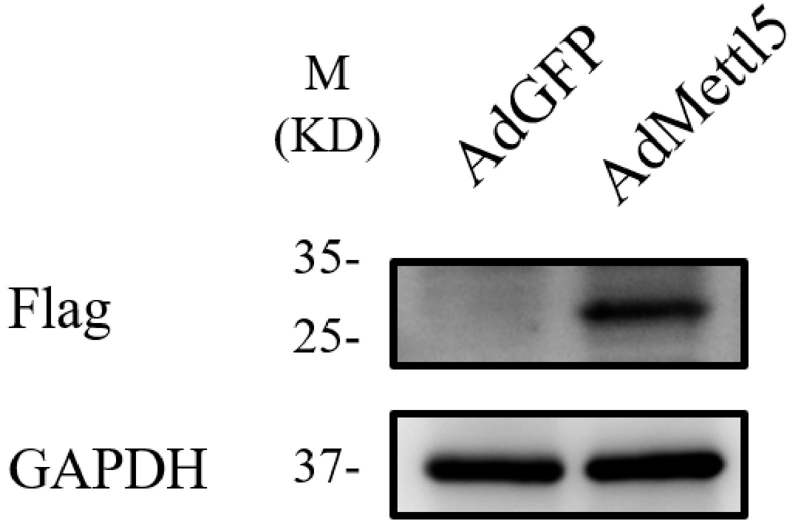

Supplemental Figure 4. Detection of overexpression of Mettl5 mediated by adenovirus in 293T. The FLAG-tagged fusion Mettl5 and GAPDH (control) are detected with specific antibodies.

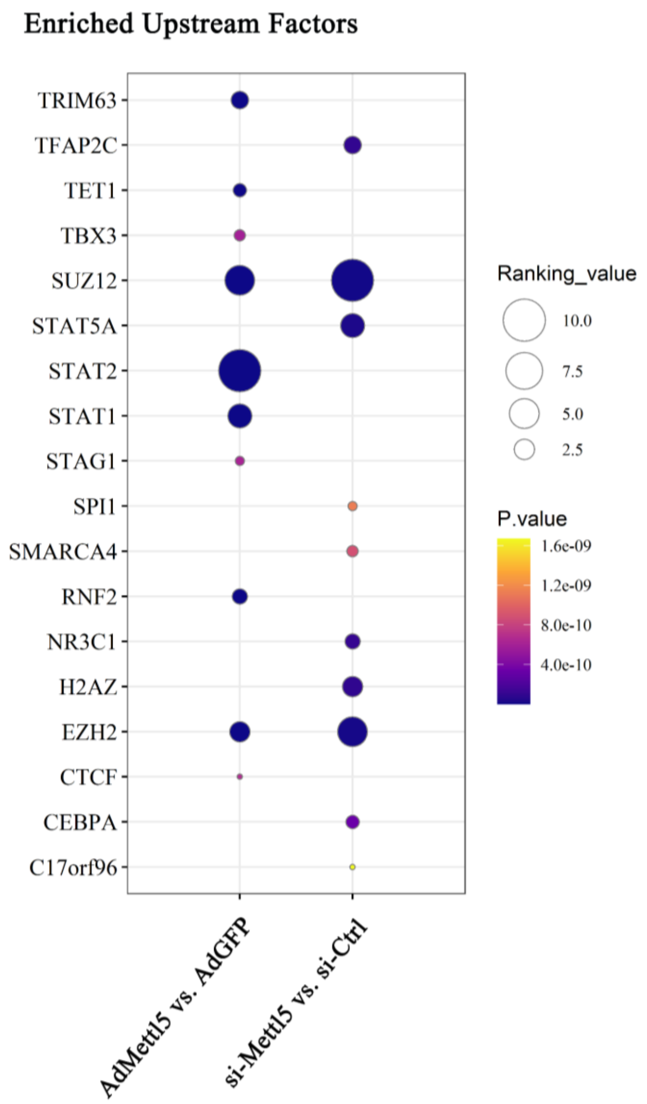

Supplemental Figure 5. SUZ12 is a critical upstream regulator mediating the METTL5-related cardiac phenotype. Upstream factor analysis by Lisa of the top 500 dysregulated genes (|Log2FoldChange|>1,adjusted P value< 0.05; ranking by adjusted P value)in si-Mettl5 vs.si-Ctrl and AdMettl5 vs. AdGFP (all under PE treatment). The top 10 upstream factors are selected ranking by p-value in each group.
